# PLASMe: a tool to identify PLASMid contigs from short-read assemblies using transformer

**DOI:** 10.1093/nar/gkad578

**Published:** 2023-07-10

**Authors:** Xubo Tang, Jiayu Shang, Yongxin Ji, Yanni Sun

**Affiliations:** Department of Electrical Engineering, City University of Hong Kong, Tat Chee Avenue, Kowloon, Hong Kong SAR, China; Department of Electrical Engineering, City University of Hong Kong, Tat Chee Avenue, Kowloon, Hong Kong SAR, China; Department of Electrical Engineering, City University of Hong Kong, Tat Chee Avenue, Kowloon, Hong Kong SAR, China; Department of Electrical Engineering, City University of Hong Kong, Tat Chee Avenue, Kowloon, Hong Kong SAR, China

## Abstract

Plasmids are mobile genetic elements that carry important accessory genes. Cataloging plasmids is a fundamental step to elucidate their roles in promoting horizontal gene transfer between bacteria. Next generation sequencing (NGS) is the main source for discovering new plasmids today. However, NGS assembly programs tend to return contigs, making plasmid detection difficult. This problem is particularly grave for metagenomic assemblies, which contain short contigs of heterogeneous origins. Available tools for plasmid contig detection still suffer from some limitations. In particular, alignment-based tools tend to miss diverged plasmids while learning-based tools often have lower precision. In this work, we develop a plasmid detection tool PLASMe that capitalizes on the strength of alignment and learning-based methods. Closely related plasmids can be easily identified using the alignment component in PLASMe while diverged plasmids can be predicted using order-specific Transformer models. By encoding plasmid sequences as a language defined on the protein cluster-based token set, Transformer can learn the importance of proteins and their correlation through positionally token embedding and the attention mechanism. We compared PLASMe and other tools on detecting complete plasmids, plasmid contigs, and contigs assembled from CAMI2 simulated data. PLASMe achieved the highest F1-score. After validating PLASMe on data with known labels, we also tested it on real metagenomic and plasmidome data. The examination of some commonly used marker genes shows that PLASMe exhibits more reliable performance than other tools.

## Graphical Abstract

**Graphical Abstract:**
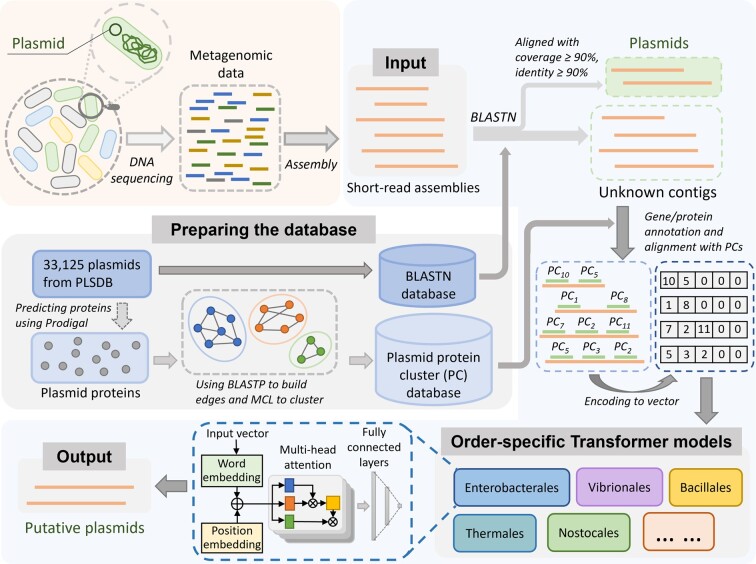

## INTRODUCTION

Plasmids are extrachromosomal mobile genetic elements capable of self-replication. Many bacteria and archaea carry plasmids, which are generally small and circular. They contain genes encoding plasmid core functions, such as conjugation transfer, replication, and partitioning (1). And they also carry accessory genes that can help hosts adapt faster to unfavorable environments, such as genes for antimicrobial resistance (AMR) (2), virulence (3), as well as plant-related nitrogen fixation and root nodulation (4). These accessory genes can be transferred horizontally to other hosts (5) and thus can spread AMR genes among pathogens, which greatly threaten human health (6). Studying the plasmids can shed light on understanding how bacteria obtain antibiotic resistance traits.

A fundamental step in elucidating the properties and functions of plasmids is plasmid identification. Because assembly programs often produce contigs, it becomes difficult to determine whether a contig originates from a plasmid or a chromosome. This problem is particularly grave for plasmid detection in metagenomic data (7). Due to the complexity of the samples, metagenomic assembly programs can produce a large number of contigs of different lengths, posing a great challenge for plasmid detection. Usually, the percentage of plasmid sequences in metagenomic data is quite small. To better study the plasmids in various samples, plasmid enrichment protocols are available for more comprehensive plasmid sequencing (8). For example, Kav *et al.* (9) used deoxyribonuclease to degrade linear DNA fragments and DNA polymerase to amplify circular DNA molecules. However, chromosomal DNA contamination is inevitable despite plasmid enrichment (10,11). Thus, there is still a great need to identify plasmid contigs from heterogeneous data.

There are two major challenges for plasmid identification. First, plasmids exhibit high genetic diversity (12). Besides frequent mutations (13), plasmids undergo large-scale structural changes during evolution, such as insertions, deletions, and translocations (14). These rapid mutations and structural changes can result in very low sequence similarity between plasmids (15). The second challenge for plasmid identification is the shared genes or segments between plasmids and chromosomes. Gene transfer frequently occurs between plasmids and chromosomes during their co-evolution (16). As a result, plasmids and chromosomes can share highly similar regions, making plasmid screening difficult, especially for short contigs. For example, Wang *et al.* studied gene transfer in 2635 *Enterobacteriaceae* and found that plasmids and chromosomes shared 33% of the 416 antibiotic resistance genes (17).

A series of tools to distinguish plasmids from chromosome contigs have been developed and can be roughly divided into three main categories: graph-based, alignment-based, and learning-based tools. Graph-based tools (18–21) attempted to reconstruct plasmids in the underlying assembly graph. These tools rely on read coverage and cyclic topology for plasmid assembly, which is best used to find relatively complete plasmids rather than short contigs.

Alignment-based tools (22–24) determine whether queries are plasmids based on the similarities between queries and references. PlasmidFinder (22) uses replicon genes, which control plasmid replication, as the reference database. Although it achieves high precision, it misses plasmid fragments without replicons. MOB-recon (23) and Platon (24) use more comprehensive databases to improve sensitivity, including plasmid sequences and marker sequences such as replicons and relaxases. Besides alignment, MOB-recon also evaluates the confidence by calculating the aligned coverage of the reference, and Platon computes replicon distribution bias to identify plasmids. These tools typically employ rigorous detection cutoffs in alignment, leading to high precision at the cost of sensitivity. Thus, alignment-based programs can exhibit a low recall in identifying highly diverged plasmids.

Learning-based methods (11,25–27) provide a promising alternative for detecting more diverged plasmids via learning abstract patterns beyond sequence similarity. Among them, cBar (25), PlasFlow (11), and PlasClass (27) used k-mer frequency as the features and applied sequential minimal optimization, fully connected neural network, and logistic regression models to predict plasmids, respectively. PPR-Meta (26) encodes the sequences and the contained codons into one-hot matrices and then trains a CNN model for prediction. These learning-based tools exhibit decreased precision on short contigs, whose length limits the capacity of feature learning. Moreover, these tools can have ambiguous predictions for contigs from shared regions between plasmids and chromosomes.

In order to achieve an optimal trade-off between sensitivity and precision, some hybrid methods combine homology search and machine learning to identify as many plasmids as possible while maintaining high precision. plasmidVerify (20), PlasForest (28), and Deeplasmid (29) belong to this category. plasmidVerify trains a Naive Bayes classifier using protein domain alignment-based features (30). PlasForest trains a random forest model using homology search results as features. Deeplasmid incorporates the profile hidden Markov model (pHMM) (31) hits of predicted proteins as key components of the feature vectors and then trains neural networks for plasmid prediction. These tools maintain high precision by leveraging alignment-based features and improve sensitivity by learning sequence patterns. However, these methods also exhibit decreased accuracy on short contigs.

In this study, we developed PLASMe for identifying plasmids from assembled contigs of different lengths. This work capitalizes on integrating the strength of sequence alignment and deep learning to achieve both high recall and precision of plasmid identification. Inspired by natural language processing (NLP), we treat plasmids as a language defined on a vocabulary of proteins. Thus, we can leverage the state-of-the-art language model, Transformer, to learn the proteins’ importance and their associations for plasmid identification. In order to maximize the capacity of feature learning, we design Transformers for each order, leading to 35 Transformer-based models for finding plasmids. We rigorously tested PLASMe on the dataset containing complete and segmented plasmids, the CAMI2 (32) simulated data, and real metagenomic and plasmidome data. All the experimental results show that PLASMe achieved a better tradeoff between recall and precision than available tools. We also interpreted the biological meaning by analyzing the attention matrix learned by Transformer.

## MATERIALS AND METHODS

### Design rationale

Building upon the unique properties of plasmids, we developed a new plasmid identification tool with the following design rationale. Although plasmid identification can be conveniently formulated as a binary classification problem, the high diversity of plasmids can pose a great challenge for feature learning. Redondo-Salvo et al. evaluated the plasmid similarity by constructing a bipartite network containing the proteomes. They concluded that plasmids of the same host taxon tend to be more similar than plasmids across different host taxa (33), which motivates us to conduct plasmid detection for different taxa. Considering that the median connection ratio of plasmids of the same order can reach 80%, a significant improvement over the ratio at the class rank (<50%), we design order-specific Transformer models for plasmid detection. An alternative design of using a unified Transformer model will also be evaluated in the Results Section.

**Figure 1.**
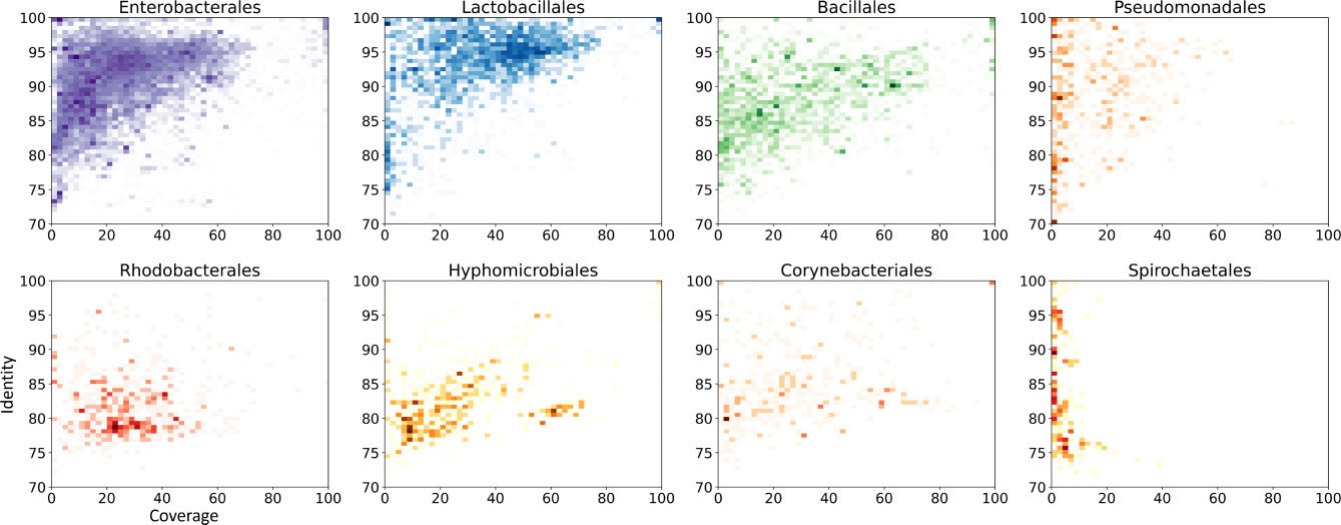
The overall coverage (x-axis) and identity (y-axis) between plasmids and chromosomes on the largest eight orders. Coverage and identity are calculated as shown in Supplementary Section 1. The similarity is shown in density scatter plots, where the darker the color, the more data points.

To tackle the second challenge of high similarities between some chromosomes and plasmids, we examined the local similarity between plasmids and chromosomes in each order via BLAST (with default *E*-value cutoff 10) and summarized the results in Figure 1. We have three observations based on the scatter plots. First, the coverage and identity of the local alignments differ significantly across different orders, further supporting our order-specific prediction models. Second, it is very rare that chromosomes and plasmids share local alignments with coverage >90%. Thus, a contig can be safely classified as a plasmid if it can be aligned with reference sequences with both identity and coverage >90%. Third, most of the hard cases of plasmids have identity >70% against chromosomes, hindering alignment programs from distinguishing them from chromosomes. Thus, PLASMe is built upon the strength of sequence alignment and Transformer, which are best used to identify closely related and diverged plasmids, respectively.

**Figure 2.**
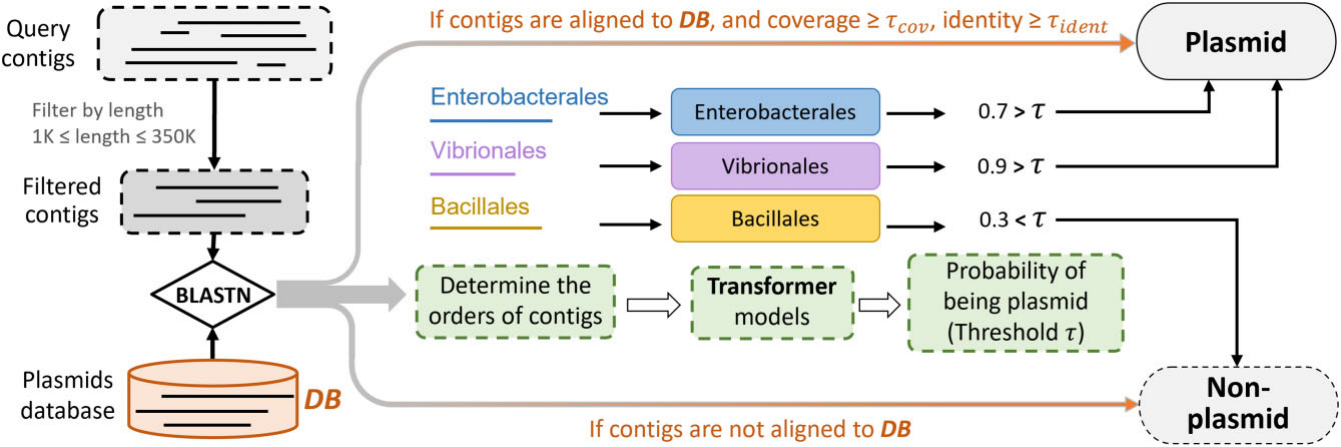
The pipeline of PLASMe. PLASMe first filters the contigs by length and aligns the filtered contigs to the plasmid database annotated with high-similarity regions. Then for the contigs aligned to the high-similarity regions with high identity and coverage, PLASMe identifies these contigs as plasmids. PLASMe determines their orders for other contigs by the alignment results and inputs them into the corresponding transformer models. And PLASMe predicts contigs as plasmids if the probability exceeds the set threshold.

The pipeline of PLASMe based on the above design is shown in Figure 2. First, we filter contigs that are shorter than 1k or longer than 350k in length. Then, we will align them against the plasmid database named *DB*, which contains 33 125 reference plasmid sequences. If the contigs are aligned with high query coverage and identity (τ_*cov*_ = τ_*ident*_ = 90% by default), they are classified as plasmids. Otherwise, they will be assigned to orders based on their alignment *e*-values (smaller than 10). Then, the order-specific Transformer will be called for plasmid prediction. If a query cannot be assigned to any order using BLAST under its default e-value, it will be classified as non-plasmids. Based on the analysis in (33), the intra-order plasmid similarity can lead to alignments with significant *e*-values. Thus, this step can help filter a large number of non-plasmids. The following sections will detail how we train the Transformer for plasmid identification.

### Transformer tokens

**Figure 3.**
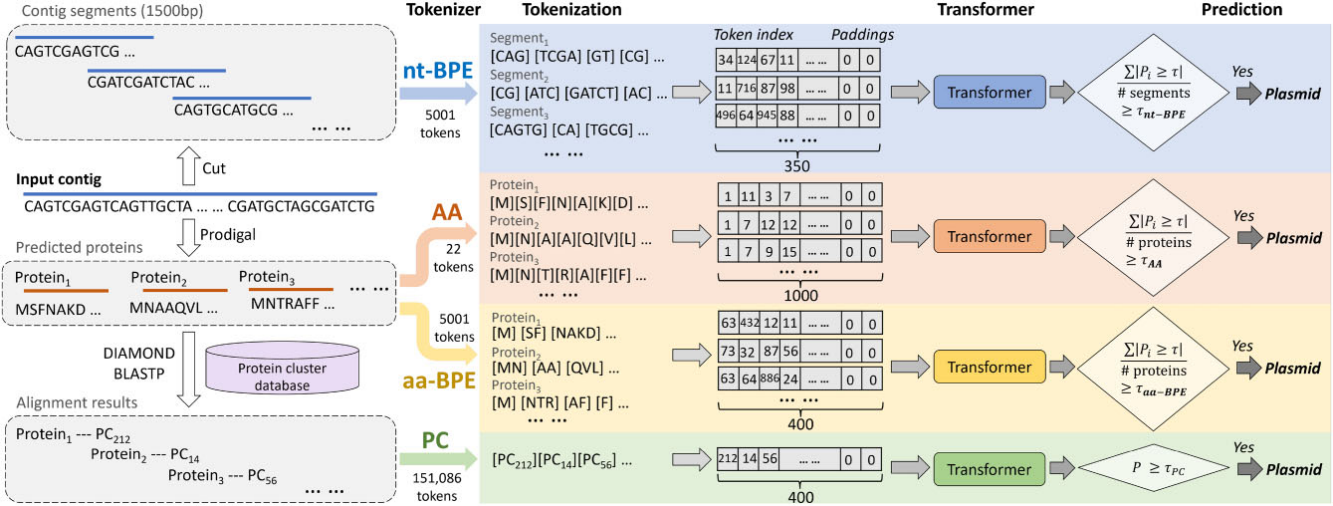
Different types of tokenizers and their corresponding prediction strategies. Input contigs are cut into segments or translated into proteins before tokenization. We implement ***nt-BPE*** on contig segments and ***AA***, ***aa-BPE*** and ***PC*** on predicted proteins to get the corresponding encoded vectors. These vectors are input into the Transformers, which will predict the probability of a contig from a plasmid. Finally, we use the majority vote on the cut segments or proteins to make a final prediction for tokens ***nt-BPE***, ***AA*** and ***aa-BPE***. And for ***PC*** tokenizer, the contig is predicted as the plasmid if the prediction probability is higher than the set threshold.

Transformer has many successful applications in the field of bioinformatics, including protein classification (34), genome or protein embedding (35,36), and molecular or protein interaction prediction (37,38). Transformer can capture the correlation between tokens and alleviate the long-time memory loss problem. However, unlike natural language, when modeling biological sequences as a language, it is not trivial to determine the best vocabulary (token set). All the proteins, amino acids or *k*-mers can be used to construct the token set and each of them has its advantages and drawbacks. In this work, we consider different sets of vocabulary and determine the best one empirically. Figure 3 shows different tokenizers and their corresponding strategies for prediction.

#### Nucleotide level

Motifs have long been used as important sequence features for DNA. They are short strings (or even regular expression patterns) defined on DNA sequence. Commonly used k-mers can be regarded as a special type of motif with fixed length k. Here, we use the Byte-Pair Encoding (BPE) (39,40) algorithm for motif-based tokenization in our ***nt-BPE*** tokenizer. The process of generating BPE tokens is shown in Supplementary Section 2. BPE allows us to use the most frequent short motifs as the vocabulary. Compared with using fixed-length k-mer as tokens, BPE can efficiently generate tokens of different lengths based on the frequency of co-occurrence of different bases. Here we trained the BPE model with all the reference plasmids, and the vocabulary size is 5002. To convert a sequence into a sentence of BPE-derived tokens, we need to limit the length of the sentence. In short, we cut the sequences into segments and convert each segment into a sentence with token length of at most 350. The detailed processing can be found in Supplementary Section 2.

#### Protein level

We also experimented with protein-based token sets, including individual amino acids, short amino acid strings (motifs), and protein clusters.

##### Individual amino acids (***AA***)

Its vocabulary contains 20 standard amino acids, undefined amino acid, and other amino acids (36,41). In our database, 98.6% of the plasmid proteins are less than 1000 aa, so we set the length of encoded vectors as 1000.

##### Amino acid subsequences (***aa-BPE***)

Similar to DNA motifs, we use BPE on proteins to build the ***aa-BPE*** tokens that include short amino acid strings with high frequency. Here, we set the length of the encoded vector to 400, which can cover 99.9% of the proteins.

##### Protein clusters (***PC***)

This token set comprises protein clusters (PCs) from plasmids. William *et al.* (29) have shown that the protein domains or curated sets of genes are more important features than the ‘physical’ features of the sequences (such as GC content). We first use Prodigal (42) to predict all the proteins from the reference plasmids and then use DIAMOND (43) to do an all-against-all alignment on all the proteins. Then we construct a graph where the nodes are proteins, and the edges represent pairwise alignment with the e-value below 1e–5. Then we apply Markov clustering (MCL) to cluster these proteins into PCs. Finally, only the clusters containing at least two proteins will be kept, leading to 151 086 PCs. We set the length of the encoded vector to 400, which can cover 99.9% of the plasmids in the database. We use the mask token (with a token ID of 0) to indicate the position of the padding and use an unknown token (with a token ID of 1) to represent the unknown proteins.

During the prediction stage, we first predict the proteins in the query sequences using Prodigal and align them to reference plasmid proteins in PCs using DIAMOND (43) BLASTP. Then we encode the vector with the index of the aligned PCs. We set stringent thresholds for the alignment of PCs, as shown in Supplementary Section 3.

### Transformer

**Figure 4.**
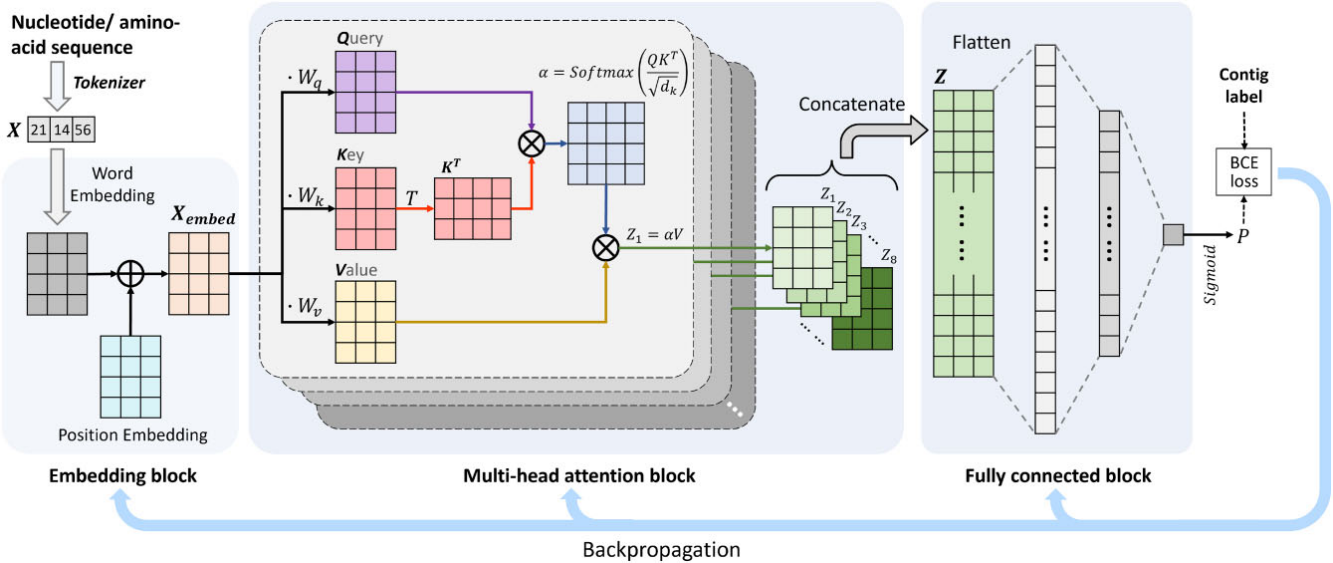
The structure of Transformer. We use the tokenizer to encode the input contig into a vector, input it into the embedding block, and then input the embedding matrix into the multi-head attention block to obtain self-attention matrices. Finally, we concatenate the attention matrices and input the flattened vector into the fully connected block for classification.

The model contains three main components: the embedding layer that converts the encoded vector into a numerical matrix, the multi-head attention block that learns the correlation information of the tokens, and the fully connected layers as the classifier for final prediction. The basic structure of the model is shown in Figure 4. The only difference between Transformers on different token sets is the length of the input vectors: 350, 1000, 400, and 400 for ***nt-BPE***, ***AA***, ***aa-BPE***, and ***PC***, respectively.

#### Embedding block

To better learn the relationship between tokens and their relative position information, the embedding block contains two parts: word embedding and position embedding. We train the word embedding to obtain the vector representation of each token and learn the representation of different positions by the position embedding. The embedding layer is similar to a lookup table, where each PC or position corresponds to its numeric vector. During training, we use the fully connected network to compute the linear projection of each token to the embedding vector. After the embedding layer, we can get *X*_*embed*_, as shown in Equation (1).


(1) \begin{eqnarray*} X_{embed}= \mathrm{WordEmbed}(X) + \mathrm{PositionEmbed}(X) \end{eqnarray*}

#### Multi-head attention block

After embedding, we feed *X*_*embed*_ into the multi-head attention block to compute the correlation between tokens. Multi-head self-attention is the integration of *h* different self-attention modules, *X*_*embed*_ is fed to each module, which computes attention matrices separately. The multi-headed mechanism allows the model to learn relevant information in different representation subspaces and thus extracts richer features. *h* is set to 8. We first multiply *X*_*embed*_ by three different weight matrices *W*_*q*_, *W*_*k*_ and *W*_*v*_ for a single attention block to obtain the query vector (*Q*), key vector (*K*) and value vector (*V*).

Then, we multiply *Q* and the transposition of *K* of all candidate positions and use softmax to compute the attention distribution α = *softmax*(*QK*^*T*^/*d*_*k*_), where *QK*^*T*^ is scaled by *d*_*k*_ and *d*_*k*_ is the dimension of *K*. Finally, we multiply α and *V* to obtain the self-attention *Z*_*i*_ as shown in Equation 2.


(2) \begin{eqnarray*} Z_i=\mathrm{softmax}(QK^T/d_k)\cdot V \end{eqnarray*}

Finally, we concatenate eight self-attention matrices *Z*_*i*_, *i* ∈ {1, 2, ..., 8} to obtain the attention-weighted feature matrix *Z* as the output of the multi-head attention block, as shown in Equation 3:


(3) \begin{eqnarray*} Z=\mathrm{concatenate}(Z_1,Z_2,...,Z_8) \end{eqnarray*}

#### Fully connected block

After obtaining *Z*, we flatten it and input it into the fully connected block, which extracts the features through two fully connected layers with dimensions 64 and 1, respectively. The output is passed through the sigmoid function to obtain the probability *P* of an input being the plasmid. We use the binary cross-entropy (BCE) as the loss function and the backpropagation algorithm to update the parameters to train the Transformer model.

### Training and prediction using different token sets

Out of the four types of tokens, only PC token-derived sentences can encode the complete input contig. Others either convert a DNA segment or a single protein into a sentence. Thus, we apply different training strategies.

During training, we cut the sequences into segments to train the ***nt-BPE*** model, and use predicted proteins to train ***AA***, ***aa-BPE*** and ***PC*** model. To improve the resolution of the ***PC*** model, we sample the sequences using sliding windows of 200, 400, 600, 800, 1000, 2000 and 4000 bp, and we use the original and sampled sequences to train the model. We also assign larger weights to a smaller class during training to avoid the influence of data imbalance. The weight is *max*(*N*_plamid_, *N*_chromosome_)/*N*_*i*_, *i* ∈ {plasmid, chromosome}.

Moreover, the final prediction for the input contig needs to consider the predictions for all the components. We use the majority vote to make the final prediction except for ***PC***. We count the proportion of segments or proteins with predicted probability greater than the set threshold τ (default 0.5) in the contig. If the proportion is greater than the threshold (τ_*nt*-*BPE*_, τ_*AA*_ or τ_*aa*-*BPE*_), we predict the contig to be the plasmid, as shown in Figure 3. When using ***PC***, we will predict the query contig as plasmid when its predicted probability is greater than τ.

### The parameters of the model and the training process

For models using different tokens, the parameters used are slightly different. Here we mainly introduce the PC model, which is used in PLASMe. The parameters used by other models can be found in Supplementary Table S3. We trained the PC model using the binary cross-entropy loss function and Adam optimizer with a batch size of 256. The learning rate and dropout rate were set to 0.001 and 0.1, respectively. Because we added many substrings for data augmentation, we found that the model can reach convergence using two epochs without overfitting. As such, we trained all models in PLASMe for two epochs. Training all models in PLASMe using the NVIDIA GeForce RTX 3090 Blower 24G graphics card took 7.3 hours. We used PyTorch deep-learning packages to build the model, and the source code has been uploaded to GitHub (https://github.com/HubertTang/PLASMe).

The model mainly consists of the Transformer’s encoder and a fully connected block. We use a single-layer encoder to transform the token vectors into a one-dimensional vector, which is then input into two fully connected layers (64, 1) for binary classification. The length of the input vector for the encoder is 400, and the embedding block inside the encoder maps each word to an embedding vector of length 512. The final output of the encoder is a one-dimensional vector of length 204,800. These model design choices were made with careful consideration of the task requirements and computational efficiency.

## RESULTS

We first compare the performance of Transformer with different tokens on the RefSeq dataset. Then, we evaluated PLASMe on multiple datasets, whose size and composition are shown in Table 1. On these datasets, we also compared PLASMe with other plasmid identification tools, including learning-based methods (cBar (25), PlasFlow (11), PPR-Mate (26), PlasClass (27)), alignment-based methods (PlasmidFinder (22), MOB-Suite (23), Platon (24)) and hybrid methods (plasmidVerify (20), PlasForest (28), Deeplasmid (29)). We evaluated the results using commonly used metrics, such as recall, precision, and *F*1-score, whose definitions are shown in Supplementary Section 4. All the benchmarked tools take contigs as input and thus can be evaluated on the same test set. For a fair comparison, we did not include graph-based plasmid assembly tools, which require different inputs and are not optimized for contigs.

**Table 1.** The size of different dataset

Dataset	# plasmids	# chromosomes
PLSDB training set	26 451	3530
PLSDB test set	6674	6674
Contig 1K test set	24 120	24 120
Contig 2K test set	23 695	23 695
Contig 3K test set	22 775	22 775
CAMI2 marine S0	1715	93 595
CAMI2 marine S1	1826	119 599
CAMI2 marine S2	2218	121 741
CAMI2 marine S3	1937	125 587
CAMI2 marine S4	1410	82 617
CAMI2 marine S5	1447	104 499
CAMI2 marine S6	1788	111 162
CAMI2 marine S7	1163	90 056
CAMI2 marine S8	1880	131 017
CAMI2 marine S9	978	99 453

### Datasets

#### PLSDB plasmids and RefSeq chromosomes

PLSDB (44) is a manually calibrated and comprehensive plasmid database. After removing plasmids shorter than 1K or longer than 350K, we keep 33 125 plasmids as *DB*. In addition, we downloaded all ‘complete’ and ‘representative’ bacterial and archaeal genomes from NCBI, yielding a total of 3775 bacterial genomes and 232 archaeal genomes. Then, we used the keywords ‘plasmids’, ‘mitochondrial’ and ‘chloroplast’ to filter out the non-chromosome sequences in these genomes. Finally, we got 4005 sequences as the reference chromosomes.

We divided this dataset into training and test sets according to their release time. The sequences published before and after the chosen date are used as training and test set, respectively. The simple method of using the same release time across different orders for data partition can lead to skewed train-vs-test ratio in different orders, which can confound the performance evaluation. Thus, we set up different dates for each order so that the training-vs-test ratio across different orders is kept similar to each other, which is roughly 4:1. The numbers of training and test sequences can be found in Supplementary Table S4. In total, the **PLSDB training set** contains 26 451 plasmids and 3530 chromosomes. And 6674 plasmids and 475 chromosomes comprise the test data. In order to mimic assembled contigs, we randomly cut chromosomes and generated a balanced **PLSDB test set** containing 6674 plasmids and 6674 chromosome fragments.

In order to test the performance on short contigs, we sampled short subsequences on the **PLSDB test set** with lengths of 1001, 2000 and 3000, respectively. Here, we choose 1001 as the minimum length because Deeplasmids requires an input length >1000. We randomly sample 5 subsequences from each sequence for each contig, leading to three test sets named **contig 1K**, **contig 2K** and **contig 3K test sets**, as shown in Table 1. During the benchmarking, we replaced the PLASMe DB database with the PLSDB training set. Therefore, there is no overlap between the training and test sets.

#### Simulated data: CAMI2 Marine dataset

Built from 1680 microbial genomes and 599 novel plasmids and viruses, CAMI2 contains multiple simulated metagenomic datasets from different ecosystems. It has been widely used to evaluate the tools for metagenomic analysis. We choose ten short-read metagenomic data simulating marine samples (**CAMI2 marine S0∼9**), because their complexity poses hard test cases for composition analysis. We used metaSPAdes (45) to assemble reads into contigs and kept contigs with lengths ranging from 1K to 350K.

#### Real data: metagenomic and plasmidome data collected from high-altitude lake

We also evaluated the performance of PLASMe on real data, including metagenomic and plasmidome data. They were collected by Perez *et al.* (46) from the same environment: Puquio de Campo Naranja, a high-altitude lake from the Andean Puna. The authors conducted plasmid enrichment for plasmidome sequencing. The metagenomic and plasmidome data contain 7 798 852 and 7 812 709 reads, respectively. After running metaSPAdes, we have 52 096 and 38 480 contigs with lengths ranging from 1K to 350K, with average lengths of 1879 bp and 1808 bp, respectively.

### Validating the rationality of the PLASMe design

We validated the rationality of our design by focusing on the following components: the order-specific model and the division of PLASMe into two stages (alignment and Transformer). First, we compared the performance of the order-specific and unified models, which is a single model trained with all training data. The results are shown in Supplementary Section 5. Supplementary Figure S1 shows that order-specific and unified models on the PLSDB test sets have comparable performance, with the order-specific model being slightly better. Supplementary Figure S2 shows that the order-specific model has observable advantages on hard test sequences that share low similarity with the training data. We also presented the performance of the order-specific model on each order in Supplementary Figure S3. Supplementary Figure S4 revealed that the model’s performance was mainly affected by the similarity between the training and test sets. The orders with lower average similarities tended to have lower *F*1-scores.

We then evaluated the performance of PLASMe at different stages, including alignment and Transformer. We also tested the performance of an alternative design that only uses alignment for taxon assignment and then uses Transformer for classification. The results are shown in Supplementary Section 7 and Supplementary Figure S5. Using only Transformer achieved good results, but the two-stage prediction had better performance on complex samples, as shown in Supplementary Figure S6.

### Performancs of different token sets

**Figure 5.**
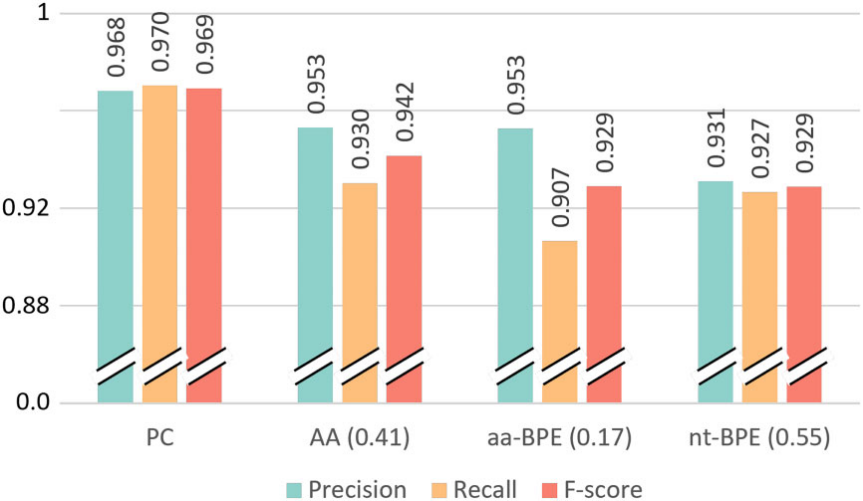
The performance of PLASMe using different token sets on the PLSDB test set. Because using ***AA***, ***aa-BPE*** and ***nt-BPE*** as token sets require the majority vote, we set different voting thresholds for them (0.41, 0.17 and 0.55, respectively) and report the best *F*1-score from different thresholds.

We first compared the performance of PLASMe with different token sets on the PLSDB test set (Figure 5).

PC-based tokens achieved the best precision, recall and *F*1-score. There are two possible reasons. First, using PCs as the tokens allows Transformer to learn the importance of proteins directly. Second, PC-based Transformer can capture the correlation between different proteins on the contig. In addition, Figure 5 shows that ***AA*** and ***aa-BPE*** performed better than ***nt-BPE***, indicating that those protein-originated features are more critical for plasmid identification. Thus, we choose PC as the default token for PLASMe.

### Performance on the PLSDB test set

**Figure 6.**
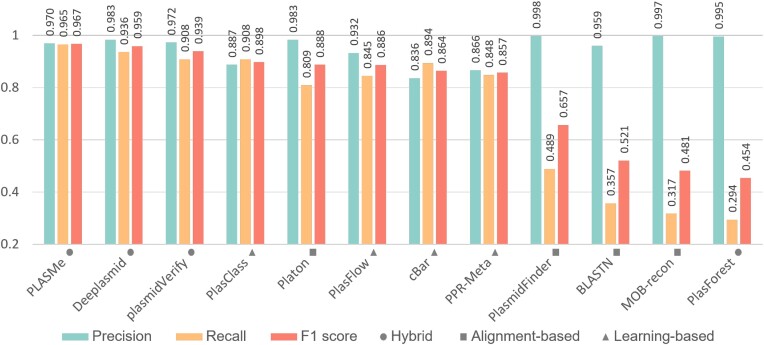
The performance of different tools on the PLSDB test set, which contains complete plasmids and segmented chromosomes. The tools are sorted from left to right by *F*1-scores. All the tools are run using their default parameters and databases except BLASTN. We used all plasmids in the PLSDB training set as the references for BLASTN, and used coverage of 60% and identity of 80% as the thresholds to identify plasmids, which are commonly used by alignment-based methods.

We used ***PC*** as the token set of PLASMe and then compared PLASMe with other tools in the PLSDB test set. The result is shown in Figure 6. PLASMe achieved the highest recall and *F*1-score. In this data, 556 (11.53%) and 4056 (84.08%) plasmids were identified by BLAST and Transformer, respectively. The top three tools (PLASMe, Deeplasmid, and plasmidVerify) are all hybrid methods that combine machine learning and features derived from protein alignments. Deeplasmid achieved the highest precision among these tools, but its recall was lower than PLASMe, leading to a lower *F*1-score. A closer looks show that Deeplasmid has difficulties recognizing short plasmid contigs. For example, we evaluated tools on test plasmids of lengths between 1000 and 3000, which account for about 5.58% of the dataset. The recall of the PLASMe is 0.937, while Deeplasmid and plasmidVerify have recalls of 0.736 and 0.528, respectively. All commands used by each tool can be found in Supplementary Table S5.

Overall, the learning-based tools achieve higher recall, and the alignment-based methods achieve higher precision. The learning-based approach can be more sensitive to remote homologous sequences by capturing sequence patterns. And the alignment-based methods usually set stringent alignment thresholds to reduce the false positive, leading to high precision but low recall. To better illustrate the results, we plot the UpSet diagrams to show the intersection of the results between different tools, as shown in Supplementary Figure S7.

Considering the small numerical differences between PLASMe and Deeplasmid in Figure 6, we generated an UpSet diagram (Supplementary Figure S8) and further analyzed the differences in their outputs, including taxonomy and relaxase type, as shown in Supplementary Table S1. The result shows that PLASMe identified more plasmids from *Enterobacterales* and *MOB*_*H*_. We also compared the performance of the two tools in different length ranges, as shown in Supplementary Figure S9. The result shows that PLASMe outperforms Deeplasmid on short contigs, while both tools exhibit comparable performance on long contigs.

To further evaluate the robustness of our approach to data partition, we performed the 5-fold cross-validation on the PLSDB dataset, as shown in Supplementary Figure S10. The overall performance (*F*1-score: 0.974±0.002) on the cross-validation is slightly better than that on the PLSDB test set (*F*1-score: 0.967), suggesting that the date-based data partition method does not lead to an easier test set for the model.

### Performance on the contig test set

**Figure 7.**
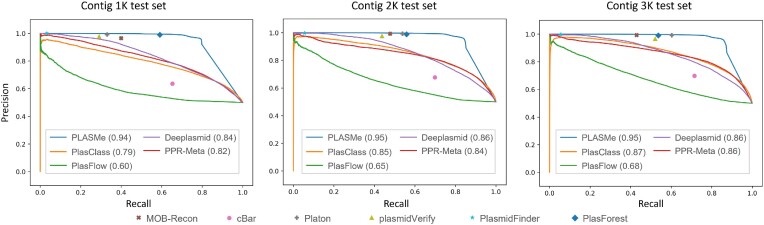
The performance on the contig test set. PLASMe, PlasClass, PlasFlow, PPR-Meta and Deeplasmid can output the predicted score, so we set the threshold to draw the PR curve. The values in brackets are the area under the curve. Other tools did not output prediction-associated scores and thus only have one data point in the figures.

In this experiment, we benchmarked PLASMe with other tools on the contig test set and drew the precision-recall curve, as shown in Figure 7. The performance of most of the tools, except BLASTN and PlasForest, improved as the contig length increased, and PLASMe achieved the maximum area under the curve (AUC). Based on the performance on the PLSDB test set, Deeplasmid and plasmidVerify were less sensitive for short sequences, which is expected.

Overall, the alignment-based approach still had higher precision and lower recall than the learning-based approach. All alignment-based tools have a precision close to 1. However, their recall on the longest contig set is at most 0.6 (by Platon). In summary, PLASMe not only achieved high recall but also maintained better precision than the existing learning-based tools. Thus, PLASMe is a more robust pipeline that can handle short contigs.

Considering that classifying short contigs may not depend much on long-term dependency, we also implemented an LSTM-based classification model for short contigs. As shown in Supplementary Figure S11, transformer still outperformed LSTM on short sequences.

### Performance on the simulated CAMI2 metagenomic datasets

**Figure 8.**
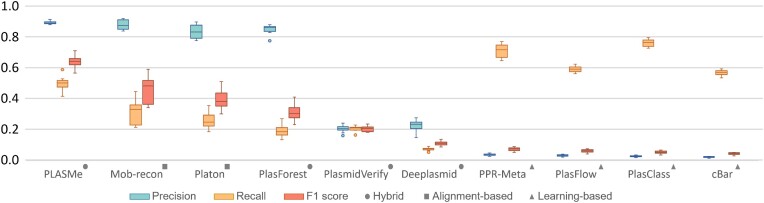
The performance on CAMI2 marine dataset. The box plot shows the results on CAMI2 marine S0∼9 (Table 1).

First, all training sequences that are identical to the test sequences are removed. To get the labels of the assembled contigs, we aligned the contigs to the references provided by CAMI2 using BLASTN. To minimize the impact of assembly errors on the performance evaluation, only contigs that can be aligned to a provided reference with both the identity and coverage above 80% are used as the test data. As shown in Table 1, the number of chromosomes is much larger than the number of plasmids in these datasets. The predicted results of different tools are shown in Figure 8. PLASMe achieves the highest *F*1-score with a good trade-off between sensitivity and accuracy.

The performance of other learning-based approaches decreases on the CAMI2 dataset compared to the **PLSDB test set**. A likely cause of their lower precision is the highly unbalanced composition of plasmids and chromosomes in the test data. This unbalance is quite representative of a typical metagenomic data, which can lead to many false positive identifications for pure learning-based approaches. In contrast, PLASMe can exclude short chromosome contigs by our alignment-based components and protein-based Transformer. Specifically, 95.76% of the chromosomes in the samples could be rejected using the alignment step in PLASMe. The hard-case contigs account for 5% of each dataset on average, and transformer achieves a precision of 77.97% and a specificity of 99.02% in the hard-case contigs. In this group of experiments, the recall of alignment-based methods is lower compared to the PLSDB test set because they set stringent thresholds in alignment to maintain high precision, but failed to recognize diverged plasmids.

As the CAMI dataset can provide assembly graphs, we also compared the performance of PLASMe with Recycler (19), which is a representative graph-based tool. The result is shown in Supplementary Figure S12. PLASMe achieves better performance. The low coverage prevented Recycler from assembling complete circular plasmids, while inconsistent chromosome coverage led to a higher number of false positives in the results. This is consistent with the analysis of Alla *et al.* (47). The average precision and recall of PLASMe were 83.1% and 76.9%, respectively, whereas those of Recycler were 63.5% and 25.9%, respectively. Further experimental details, including performance metrics, are provided in Supplementary Section 12.

### Performance on the real metagenomic and plasmidome datasets

Because we did not know the true composition of real data, it is hard to determine the labels of contigs. Therefore, we analyzed the performance by analyzing the existing markers in the contigs. We used Prokka (48) to annotate genes in the identified contigs. Then, we examined AMR and chromosome associated proteins (CAP) because they are enriched in plasmids and chromosomes, respectively. The number of AMRs and CAPs in the predicted plasmid contigs by different tools are shown in Tables 2 and 3. Different tools have highly different annotations of AMRs and CAPs. Overall, the plasmids reported by PLASMe have the highest ratio of AMR to CAP, suggesting that its finding is reasonable from this perspective. plasmidVerify identified 4 and 3 contigs containing AMR, respectively. However, it also identified five contigs containing CAP on both data, indicating that the precision of plasmidVerify is not high. Deeplasmid did not identify any contig including AMR. Furthermore, although learning-based tools predicted more contigs as plasmids, many identified contigs contain CAP. This is consistent with the observation that pure learning-based tools tend to have lower precision than alignment-based tools.

**Table 2.** The annotation on plasmid contigs identified by different tools on the metagenomic (ERR3083899) data, including the number of contigs predicted to be plasmid, and the number of contigs containing AMR and CAP

Tools^a^	# contigs	# AMR	# CAP
**PLASMe** ^b^	1544	20	2
plasmidVerify^b^	621	4	5
Deeplasmid^b^	146	0	0
PlasForest^b^	23	0	0
Platon^c^	10	0	0
PlasClass^d^	26 068	106	35
PPR-Meta^d^	18 219	76	41
cBar^d^	17 922	59	43
PlasFlow^d^	12 205	32	18

^a^ MOB-Recon and PlasmidFinder did not identify any plasmids and therefore are not shown in the table.

^b^ Hybrid method.

^c^ Alignment-based methods.

^d^ Learning-based methods.

**Table 3.** The annotation on plasmid contigs identified by different tools on the plasmidome (ERR3528510) data, including the number of contigs predicted to be plasmid, and the number of contigs containing AMR and CAP

Tools ^a^	# contigs	# AMR	# CAP
**PLASMe** ^b^	1646	19	2
plasmidVerify^b^	562	3	5
Deeplasmid^b^	383	0	0
PlasForest^b^	18	0	0
Platon^c^	10	1	0
PlasClass^d^	19 940	90	39
PPR-Meta^d^	14 880	60	36
cBar^d^	14 806	73	30
PlasFlow^d^	10 878	41	17

^a^ MOB-Recon and PlasmidFinder did not identify any plasmids and therefore are not shown in the table.

^b^ Hybrid method.

^c^ Alignment-based methods.

^d^ Learning-based methods.

To evaluate the results more comprehensively, we calculated the intersection of the results between different tools, as shown in Supplementary Figure S13. PLASMe is very sensitive in identifying these highly confident plasmid contigs. Moreover, we evaluated the running time of different tools in Table 4. PLASMe is one of the fastest tools. The running time comparison reveals a significant difference in the tools’ efficiency. We discussed the possible reasons behind this based on the method design in Supplementary Section 14.

**Table 4.** Running time (hour: minute: second) of different tools on the metagenomic and plasmidome data. Benchmarking was performed on a PC with an Intel Core i7-9700 (8 cores) CPU and GeForce RTX 2070 GPU

Tools	Metagenomic	Plasmidome
cBar	00:00:27	00:00:20
PlasFlow	00:02:45	00:01:50
PlasClass	00:03:30	00:02:34
**PLASMe**	00:03:16	00:03:06
Mob-Recon	00:17:30	00:12:57
PlasForest	00:24:09	00:17:51
PlasmidFinder	00:24:44	00:18:19
PPR-Meta	01:15:43	00:53:54
plasmidVerify	08:59:07	06:18:49
Platon	15:46:59	11:35:34
Deeplasmid	90:19:35	66:34:26

Moreover, running PLASMe also does not require much memory, consuming 6.58G and 6.49G on the metagenomic and plasmidome datasets, respectively.

### Interpreting the performance of Transformer

**Figure 9.**
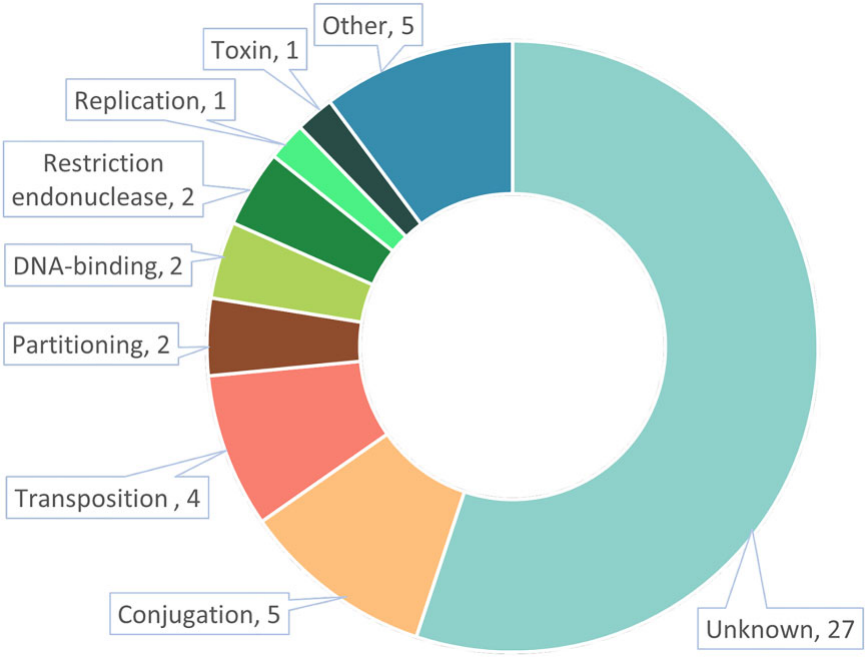
The function annotations of the top 50 important tokens.

To interpret the performance of Transformer, we evaluated the importance of ***PC*** tokens by analyzing the attention distribution. We found that the plasmids’ core genes play an essential role in Transformer’s prediction. We visualized the attention distribution for an example sequence (NCBI accession number: NC_011410.1), as shown in Supplementary Figure S14. And the detailed method of evaluating the token importance is shown in Supplementary Section 15. After obtaining the token importance, we annotated the proteins in the corresponding PCs using InterPro (49), where we took the longest protein as the representative protein and used the function of this protein as the function of the PC. We annotated the functions of the top 50 tokens and presented the function distribution in Figure 9. Among of them, 23 have protein function annotations, and 12 are related to plasmid core genes: 5 are about conjugation, 4 about transposases, 2 about partitioning and 1 about replication. Conjugation proteins are responsible for the horizontal gene transfer between bacteria, while the spread of AMR and virulence factors can help hosts improve their adaptability to the environment. Among these genes associated with conjugation, tokens annotated as TrbM (InterPro entry: IPR009989) appeared 1024 times, of which 328 were evaluated as important tokens. This family contains MpfK and its functional homologue kikA proteins, which are responsible for efficient conjugative transfer (50). Transposition is also an essential process for transferring DNA between bacterial cells. With the help of transposition proteins, transposons can be integrated into new sites of other plasmids or chromosomes. Transposase, IS116/IS110/IS902 (InterPro entry: IPR003346), a transposase required for efficient transposition of insertion sequences or transposon DNA, has appeared 1120 times as a token, of which 402 times were rated as important tokens. The detailed function descriptions of the annotated tokens are provided in Supplementary Table S6.

We plotted the histogram of the average attention score of each token in Supplementary Figure S15. The distribution implies that only a few PCs have high scores, while the majority of PCs may only contribute to specific sequences, resulting in a lower score. We also utilized a learning-based tool, DeepFRI (51), to predict the functions of unknown PCs using both sequence and AlphaFold-predicted (52) structures. The results can be found in Supplementary Table S2. The function analysis indicated that these tokens were also associated with the core functions of plasmids. This experiment shows that PLASMe can capture the proteins that play essential roles in plasmids.

## DISCUSSION

In this work, we present PLASMe, a tool combining deep learning and homology search to identify plasmids in short-read assemblies. Contigs that are highly similar to known plasmids are directly classified as plasmids. Inputs that share somewhat similarities are examined by the deep learning component in PLASMe. Specifically, we constructed plasmid protein clusters as vocabulary and then used Transformer to learn the importance of proteins and the correlation between them. As shown in the experiments, PLASMe achieved higher recall than alignment-based tools and higher precision than learning-based tools. By interpreting Transformer, we found that the more important tokens for identifying plasmids were associated with plasmid core genes. Overall, PLASMe can quickly and accurately identify plasmids in assembled contigs.

Based on our experimental results, short contigs tend to contain more false positives. Due to the horizontal gene transfer between chromosomes and plasmids, identifying plasmids from short sequences may result in false positives. Thus, identifying fragmented plasmid sequences remains a challenging task. Despite the challenge, the experimental results showed that PLASMe had the best performance on identifying short plasmids. For example, as demonstrated in Figure 7, PLASMe demonstrates high precision on data with contig lengths of 1k, 2k, and 3k, with precision reaching 0.92, 0.97 and 0.98 at the recall of 0.8, respectively. Moreover, the average length of contigs in CAMI datasets is only 2807 bp, and PLASMe achieves a precision of 0.92. Moreover, PLASMe will output the identified plasmids with marking the starting and ending position in an input sequence if it can be aligned to chromosomal genomes with high similarity. Users can use our predictions and the marked regions to further screen false positives using their domain knowledge about the data/samples. Detailed information can be found in Supplementary Section 18.

Compared to *k*-mer, BPE, and character-level tokenization, PC-level token is better at learning the importance of different proteins and the relationships between them. Especially for longer sequences, using the proteins as tokens and embedding them into vectors results in much shorter vectors than those generated by k-mer or BPE. Using shorter input vectors can reduce the model’s complexity (fewer parameters), which helps model training on a GPU. Thus, under limited computational resources, using a PC-level tokenizer can better integrate information from longer sequences. However, PC-level tokenization also has some limitations, as it is more prone to out-of-vocabulary (OOV) issues. Therefore, for newer plasmids, using PC tokens may lead to false negatives.

There are a few problems left for future work. First, because plasmids have a large diversity, novel plasmids may contain proteins that cannot be aligned with the current database, leading to inaccurate predictions. If a contig only contains new proteins that cannot be aligned to the database, it poses a difficult case for most tools. Specifically, PLASMe and alignment-based tools cannot identify these contigs because there is no alignment. Deeplasmid also relies on alignment-based features such as those from HMMER. Without alignment, it can only use features such as sequence length and the number of contained genes, leading to inaccurate predictions. Tools that rely on motifs or k-mer frequency, such as PPR-Meta and PlasFlow, can only predict accurately if the sequences contain specific motifs or k-mer frequency distributions that have been seen before. Another situation is that plasmids contain ‘unseen’ proteins that can still be aligned with known ones. PLASMe can still accurately classify these contigs as long as they possess known essential proteins. However, alignment-based tools may classify these contigs as chromosomes due to the poor alignments. For example, Deeplasmid may not construct Pfam vectors correctly for these novel proteins, jeopardizing its overall performance. To improve the model’s sensitivity, besides updating and expanding the database in time, we will dig deeper into the relationship between plasmid proteins to build a bridge between known and unknown tokens.

Second, the current tokens contain only proteins of plasmids. Studies have shown that proteins critical for bacterial survival are more likely to be found in chromosomes (53). Therefore, adding proteins specific to chromosomes may help further improve the learning model’s accuracy. We will add the essential chromosome proteins to the current protein database to improve precision further. Third, the interpretation of the Transformer identified potentially unannotated PCs that may also play an important role in the life of plasmids. Predicting the functions of these unannotated plasmid proteins may help study the evolutionary as well as ecological significance of plasmids.

## Supplementary Material

gkad578_Supplemental_File

## Data Availability

PLASMe is implemented in Python, which can be downloaded at https://github.com/HubertTang/PLASMe (permanent DOI: http://doi.org/10.5281/zenodo.8068454). The accession number of metagenomic and plasmidome data are ERR3083899 and ERR3528510, respectively.
