# Supplementary material for: PLASMe: a tool to identify PLASMid contigs from short-read assemblies using transformer
Source: Nucleic Acids Res. 2023 Jul 10;51(15):e83. doi: 10.1093/nar/gkad578 (PMC10450166; doi:10.1093/nar/gkad578)
Supplement: gkad578_Supplemental_File [file gkad578_supplemental_file.pdf]

PLASMe: a tool to identify PLASMid contigs from short-read assemblies using Transformer

Xubo Tang, Jiayu Shang, Yongxin Ji, and Yanni Sun

Supplementary materials

Contents

1 Evaluating similarity between plasmids and chromosomes **2**

2 Applying nt-BPE on the nucleotide sequences **2**

3 Thresholds of PC tokens **2**

4 Evaluation metrics **3**

5 Comparing the performance and computational costs between order-specific and unified models **3**

6 Comparing the performance on different orders **4**

7 Evaluating the performance in different stages **5**

8 The UpSet diagrams of the identified plasmids between different tools on the PLSDB test set **7**

9 Comparing the performance of PLASMe and Deeplasmid on the PLSDB test set **8**

10 5-fold cross-validation on the PLSDB dataset **9**

11 Comparison between Transformer and LSTM **10**

12 Comparing PLASMe and Recycler on the CAMI datasets **11**

13 The intersection between different tools on the real data **12**

14 Running time analysis of different tools **13**

15 Method of extracting important tokens **13**

16 The distribution of the average attention score of each token **14**

17 Predicting protein functions of the unknown tokens **14**

18 Annotating the high-similarity regions on the plasmids **15**

19 Supplementary Tables **15**

1 Evaluating similarity between plasmids and chromosomes

During the evolutionary process, plasmids and chromosomes undergo gene transfer, which results in sharing similar regions between their sequences. We aligned plasmids with chromosomes using BLASTN and calculated query coverage and identity to demonstrate the gene transfer between them. A query may have several alignment regions $S = s_1, s_2, ..., s_n$, and the region s_i may be aligned to multiple subject sequences. Therefore, we keep only the results with the maximum bit score for overlapping regions. When calculating the coverage, we divide the total length of the query by the length of all alignment regions on the query, as shown in Eqn. 1. When calculating the identity, we calculate the weighted identity of different regions based on their lengths, as shown in Eqn. 2.

$$\text{coverage} = \frac{\sum \text{length}(s_i)}{\text{length}(query)} \tag{1}$$

$$\text{identity} = \frac{\sum identity_{max\ score}(s_i) * length(s_i)}{\sum \text{length}(s_i)} \tag{2}$$

2 Applying nt-BPE on the nucleotide sequences

The basic idea of applying BPE in biological sequences is to count the most frequent nucleotide combinations in the corpus. To build the vocabulary, BPE will start with single nucleotides (A, T, C, G) and gradually expand the vocabulary by adding DNA substrings with high occurrence frequency. BPE will iterate and repeat the above operation to synthesize longer tokens until the target vocabulary size is reached.

During the encoding process using nt-BPE, we cut the plasmids into segments of length 1500bp to limit the length of the encoded vectors. Then we use the trained BPE model to encode the segments into vectors that contain 350 tokens. For the encoded vectors that contain more than 350 tokens, we keep only the first 350 tokens, while for vectors that contain less than 350 tokens, we will pad the zeros afterward until the length reaches 350. We input all the segments from the training database to train the model. For testing, because each contig will be cut into 1500bp segments, we take a majority vote on the results of all segments to predict whether the contig is a plasmid.

3 Thresholds of PC tokens

To obtain the threshold of each $\boldsymbol{PC}$ token, we did the all-against-all alignment of the proteins inside each PC and aligned all plasmid proteins with chromosome proteins. Then we set the thresholds on the identity. We set the identities within proteins in PC_i to I_{p_i}, and between proteins in PC_i and chromosomes to I_{c_i}. If $min(I_{p_i}) \leq max(I_{c_i})$, we discard PC_i and finally leave 145,400 PCs as tokens of the Transformer, occupying 96.2% of the total PCs. As shown in Eqn. 3, we set the threshold t for each PC.

$$\text{t} = \begin{cases} min(min(I_{p_i}), 80), & if\ I_{c_i} = empty \\ (min(I_{p_i}) + max(I_{c_i}))/2, & if\ min(I_{p_i}) \geq max(I_{c_i}) \\ Youden_J(I_{p_i}, I_{c_i}), & if\ min(I_{p_i}) < max(I_{c_i}) \end{cases} \tag{3}$$

As shown in Eqn. 3, when PC_i does not hit any chromosomes, because there are some small PCs in which the protein similarity is very high and $min(I_{p_i})$ to be close to 1, we set the threshold to $min(min(I_{p_i}), 80)$ in this case to improve the sensitivity. When $min(I_{p_i}) \geq max(I_{c_i})$, we set $(min(I_{p_i}) + max(I_{c_i}))/2$ as the threshold value in order to balance sensitivity and precision. When $min(I_{p_i}) < max(I_{c_i})$, we use Youden's J index [1] to calculate the threshold. This measure combines sensitivity and specificity, usually defined as $J = \text{sensitivity} + \text{specificity} - 1$. We calculate the threshold t of J_{max} by computing $J_{max} = max(\text{sensitivity}(t) + \text{specificity}(t) - 1)$.

4 Evaluation metrics

Following the previous works, we calculate the precision, recall, and F1-score as the performance metrics:

$$\text{Precision} = \frac{TP}{TP + FP} \tag{4}$$

$$\text{Recall} = \frac{TP}{TP + FN} \tag{5}$$

$$\text{F1 score} = \frac{2 * \text{Precision} * \text{Recall}}{\text{Precision} + \text{Recall}} \tag{6}$$

where TP, FP, FN are the number of true positive, false positive, and false negative predictions, respectively.

5 Comparing the performance and computational costs between order-specific and unified models

Considering the high diversity of plasmids, we trained models for each plasmid order. To show the advantages of this method, we trained a unified model using the PLSDB training data and compared its performance with order-specific models on the PLSDB test set and contig 1K/2K/3K test sets. To train the unified model, we used all the training sequences from all the orders to train a single model. The results are shown in Figure S1.

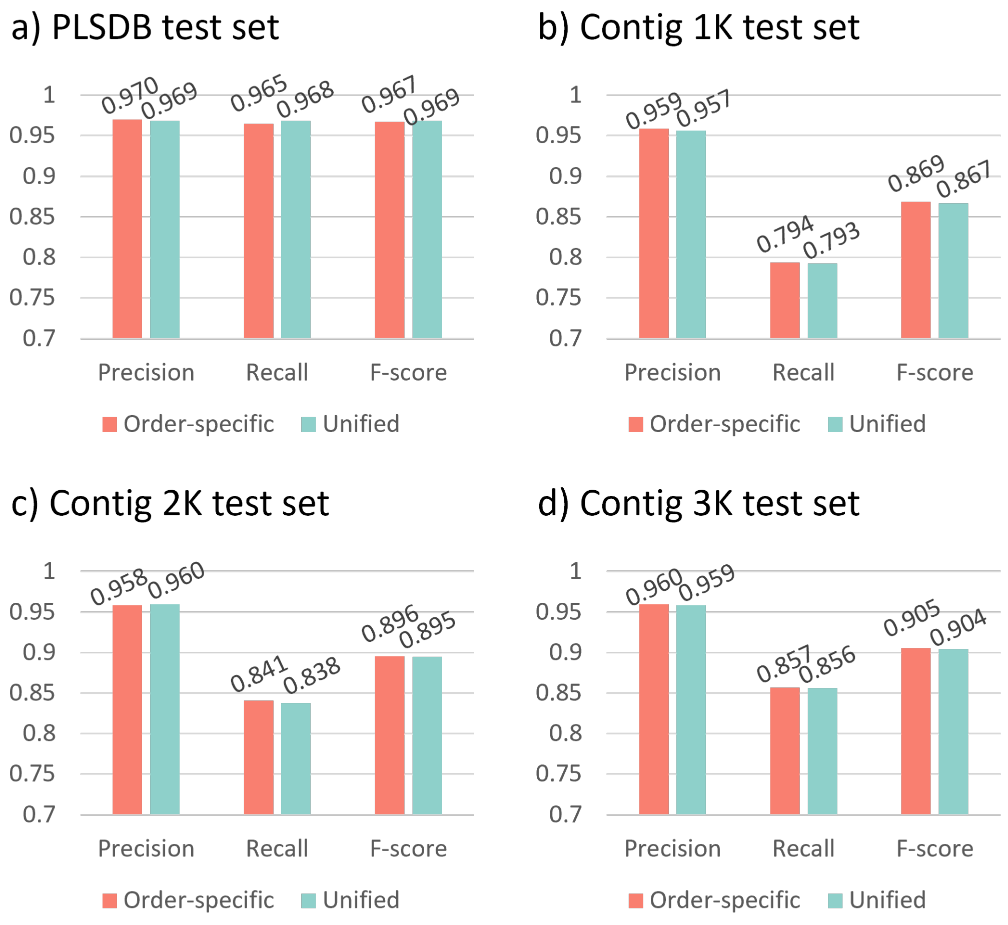

Figure S1: The performance of order-specific and unified models on different test sets. a) The PLSDB test set. b) Contig 1K test set. c) Contig 2K test set. d) Contig 3K test set.

As shown in Figure S1, the comparison shows that these two pipelines yield similar results on the PLSDB/contig datasets, with the unified model 0.1% lower in F-1 scores. However, a closer look shows that the two models' performance mainly differs in the test sequences that tend to be misclassified. Specifically, we extracted the plasmid contigs that have low similarity with the training set (alignment e-value greater than 10) and chromosome contigs that can be aligned with training plasmids with coverage/identity above 50%. We build the hard 1K, hard 2K, and hard 3K test sets from the contig 1K, contig 2K, and contig 3K test sets. The performance of the order-specific model and the unified model is shown in Figure S2.

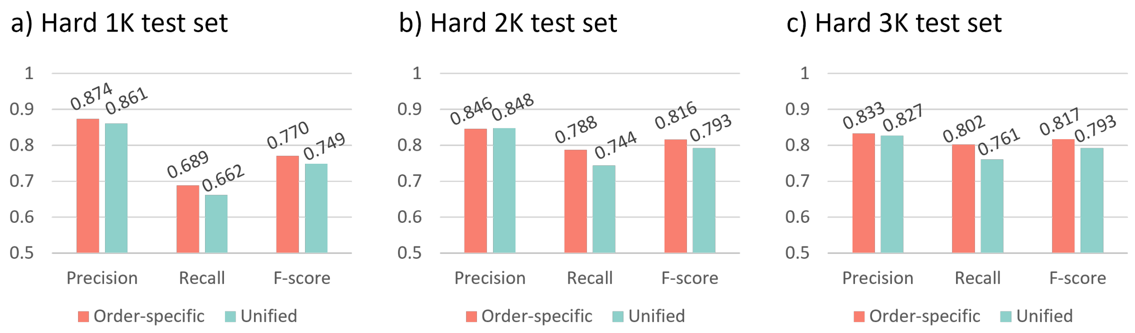

Figure S2: The performance of order-specific and unified models on the hard-case samples.

On the hard-case datasets, we can see that order-specific models still perform better than unified models. As the main usage of plasmid detection is to find the hard cases that tend to be missed by alignment-based methods, we choose to use the order-specific model.

In addition to model performance, another consideration is the computational costs of the two methods. During the training process, these two methods have almost no difference in computational resource usage and time cost because the training data size is the same. Specifically, the unified model uses all the data to train a single model. In contrast, the order-specific model divides all the data into 35 parts and trains the model using the corresponding subset. On the PLSDB training set, training the order-specific models took 7h20min, while training the unified model took 7h14min. During the testing stage, these two methods still do not differ much in memory, but there is a slight difference in running time. For example, when predicting the PLSDB test set, the Transformer in the unified model took 7.6s, while the order-specific model took 40.4s. The extra time comes from the overhead of switching models based on the prediction of the orders. Since the model is switched at most 34 times during prediction, the order-specific model takes at most 32.8 extra seconds regardless of the number of input sequences.

6 Comparing the performance on different orders

We used the PLSDB test set and fed the testing contigs to the corresponding orders (models) to evaluate the performance of PLASMe on different orders. The results are shown in Figure S3, which shows that the performance of PLASMe on some orders is worse than on others. Specifically, the differences in precision are relatively small, while the differences in recall are larger. Therefore, we hypothesize that sequence similarity between the test set and training set may account for this observation, with lower similarities leading to lower recall. To test this hypothesis, we used Dashing [2] to calculate the similarity between the test set and training set under different orders. We plotted the relationship between precision and recall against average similarity, as shown in Figure S4. We only colored the orders with average similarity below 0.2 (other orders are left with gray). According to the right panel, orders with lower average similarities tend to have lower recalls.

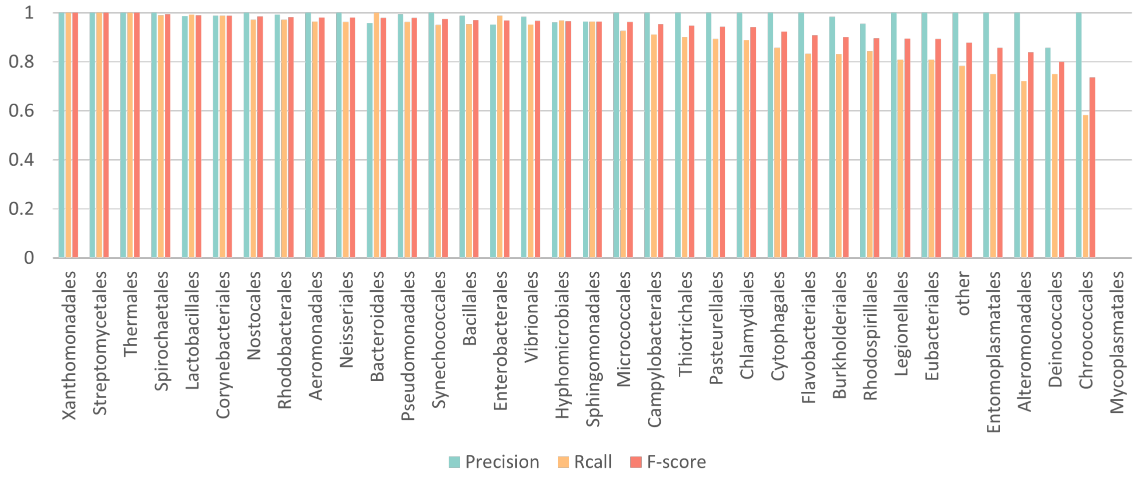

Figure S3: The performance of PLASMe on different orders.

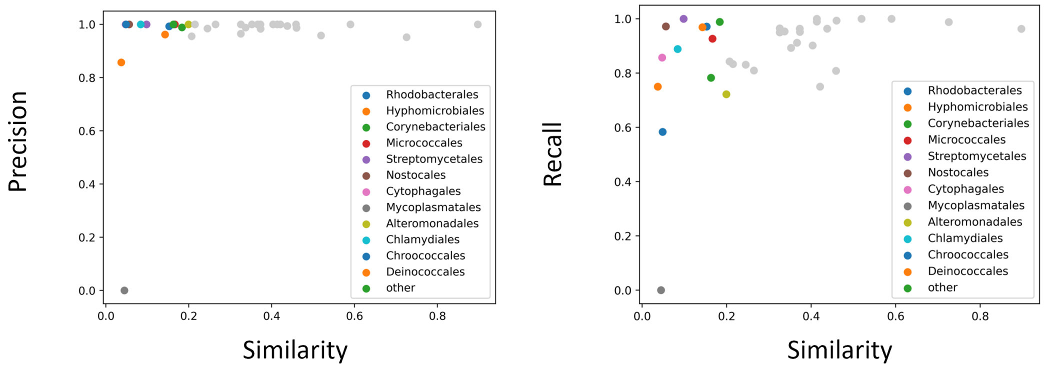

Figure S4: The correlation of precision/recall with intra-order similarity.

7 Evaluating the performance in different stages

PLASMe identifies some plasmid contigs by alignment with the stringent threshold and then uses Transformer to annotate the contigs that BLASTN cannot identify. We first analyzed the model's performance in both the alignment and transformer stages. Then, we tested a modified PLASMe that only used the alignment for taxonomic assignment and assigned predictions based solely on the order-specific Transformer models. We used the PLSDB, contig 1K, contig 2K, and contig 3K test sets to evaluate the performance. The results are shown in Figure S5.

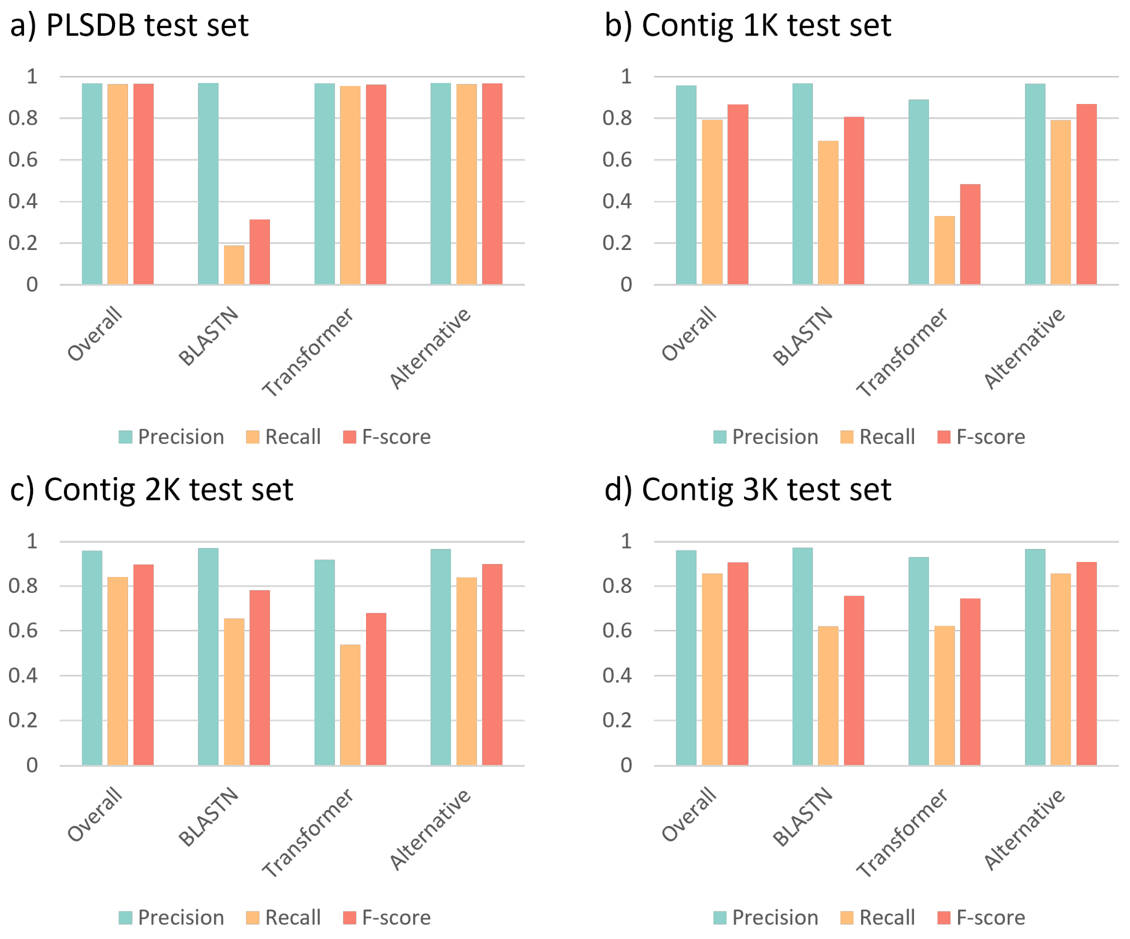

Figure S5: The performance of the model at different stages in PLASMe. ***Overall***: the overall performance of PLASMe. ***BLASTN***: the performance of PLASMe's BLASTn stage. ***Transformer***: the performance of PLASMe's Transformer stage, which takes the test sequences after running BLASTn. ***Alternative***: the performance of the alternative design that uses BLAST to assign taxa and then classifies using Transformer.

As shown in Figure S5, we examined the model's performance at different stages. We first use BLASTn to identify plasmids, and then input contigs with identity and coverage less than 90 to Transformer to classify them. When evaluating the results of the different stages, we used all the sequences in the BLASTn stage, while in the Transformer stage we used the remaining sequences after the BLASTn prediction. We can see that on the PLSDB test set, BLASTN has higher precision but lower recall, which is expected. And the Transformer classifies the rest of the sequences with a high F-score. On the contig test sets, as the contigs' length increases, the BLAST's precision remains almost the same while the recall decreases. This is also expected because of the stringent thresholds set in the alignment, which becomes increasingly difficult to meet for longer sequences. Additionally, longer sequences typically contain more proteins, leading to increased precision and recall of the Transformer.

Furthermore, we conducted a more detailed analysis of the alternative design "Alternative", which assigns taxa using alignment and then predicts plasmid using Transformer. We observed that if the classification was performed solely using Transformer, the performance was almost equivalent to that achieved using both alignment and Transformer. It suggests that Transformer can achieve classification results consistent with alignment on highly similar sequences. To demonstrate that combining alignment with Transformer gives better results than using only Transformer for prediction, we compared the performances on the CAMI datasets, which are more complex datasets. The results are shown in Figure S6. We can see that combining alignment and Transformer can achieve better results.

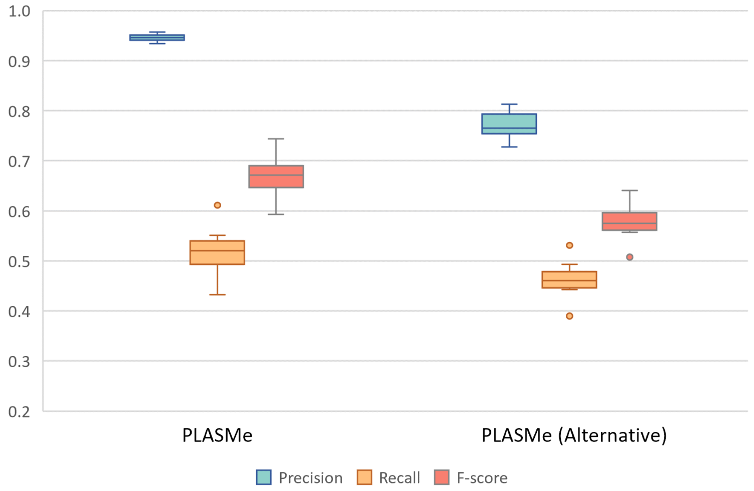

Figure S6: The comparison of PLASMe and PLASMe (Alternative) on CAMI marine datasets.

8 The UpSet diagrams of the identified plasmids between different tools on the PLSDB test set

To further compare the results of the different tools on the PLSDB test set, we further computed the intersection of the results between different tools, as shown in Figure S7.

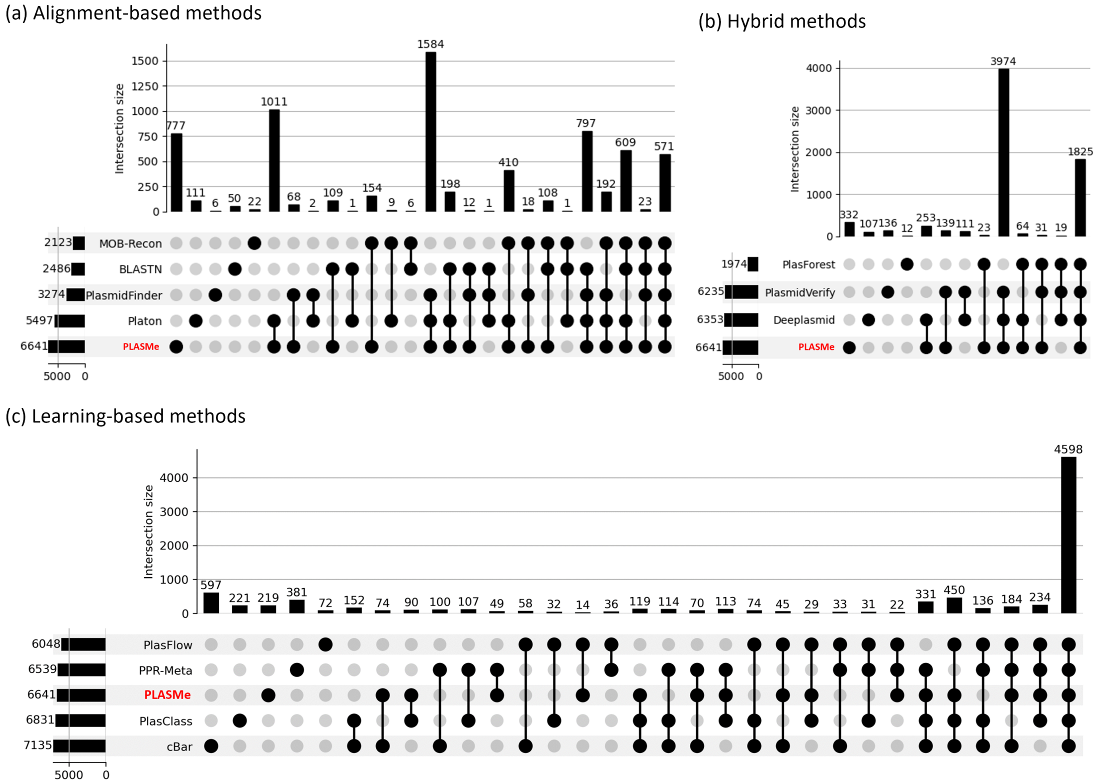

Figure S7: The intersection of the identified plasmids between different tools on the PLSDB test set. We compared PLASMe with alignment-based, learning-based, and hybrid tools, respectively.

PLASMe exhibited higher sensitivity than alignment-based methods, thus we can observe more uniquely identified plasmids. As demonstrated in Figure S7 (b), most contigs are shared among PLASMe, Deeplasmid, and PlasmidVerify, whereas PlasForest identified fewer plasmids. For the comparison with learning-based methods, the largest set is the intersection of all the tools, containing 4598 contigs.

9 Comparing the performance of PLASMe and Deeplasmid on the PLSDB test set

We further analyzed the meaningful biological difference between PLASMe and Deeplasmid on the PLSDB test set. First, we plotted an UpSet diagram to compare their results, as shown in Figure S8.

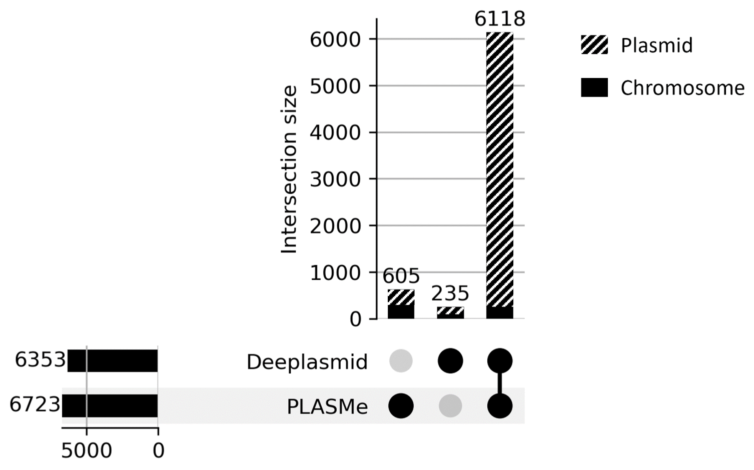

Figure S8: The UpSet diagram of Deeplasmid and PLASMe on the PLSDB test set. The number above the bar is the size of the intersection.

In Figure S8, we observe that PLASMe and Deeplasmid identified 6,723 and 6,352 contigs as putative plasmids, where 6,118 contigs were commonly identified by both tools. PLASMe and Deeplasmid identified 605 and 235 distinct contigs, respectively, of which 336 and 135 contigs are plasmids. Thus, the differences in the identified plasmid contigs by the two tools mainly lie in the 336 and 135 contigs. We conducted a comparative analysis from three perspectives in Table S1.

- Length
- The proportion of contigs aligned with the PLASMe training plasmids
- The taxonomic analysis and the relaxase type

As shown in Table S1, PLASMe can identify a greater number of short plasmids. We then aligned the identified contigs to the training plasmid sequences and observed that some plasmid contigs uniquely identified by Deeplasmid cannot be aligned to our training data, which explains why these contigs are missed by PLASMe. Moreover, we analyzed the taxon of identified distinct plasmids. We found that PLASMe identified more contigs from Enterobacterales, while Deeplasmid identified more contigs from "other" order, which combines small orders. This is expected because these orders are very small and thus lacks sufficient training data for PLASMe. To further compare the biological differences, we also used MOB-Suite to predict the relaxase type of identified plasmids. We found that PLASMe could find more MOB_H, while Deeplasmid could find more MOB_P.

Considering that the sequence length affects the performance of the tools, we compared their performance on their identified putative plasmids with different length ranges, as shown in Figure

Table S1: The summary information about the results of PLASMe and Deeplasmid.

Tools	Median length (bp)	The proportion of contigs that can be aligned with the PLASMe training plasmids (#)	The top 3 taxonomy (#)	The top 3 Relaxase type (#)
PLASMe (336 plasmid contigs)	11,031	98.5% (331)	Enterobacterales (83), Bacillales (30), Pseudomonadales (19)	MOB_H (14), MOB_C (12), MOB_V (8)
Deeplasmid (135 plasmid contigs)	14,837	20.0% (27)	other (31), Bacillales (16), Mycoplasmatales (14)	MOB_P (17), MOB_Q (8), MOB_V (4)

S9. The recall of PLASMe is always higher than Deeplasmid at different lengths, and the difference between the two is bigger for shorter sequences. And the precision of PLASMe is slightly lower, but overall, PLASMe achieves higher or similar F-score at different lengths. Hence, it can be concluded that PLASMe outperforms Deeplasmid on short contigs, while both tools exhibit comparable performance on long contigs.

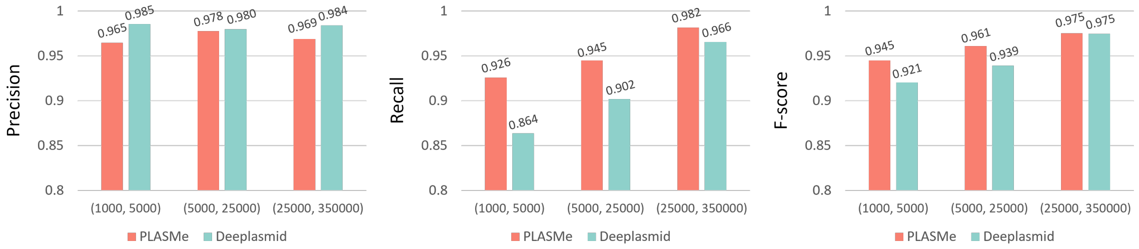

Figure S9: The performance of PLASMe and Deeplasmid on inputs with different sequence lengths ranging from 1000 to 5000 bp, 5000 to 25,000 bp, and 25,000 to 350,000 bp.

10 5-fold cross-validation on the PLSDB dataset

To further evaluate the robustness of our approach to data partition, we performed the 5-fold cross-validation on the PLSDB dataset. For each order of plasmids and chromosomes, we divided them into five parts each and then performed a 5-fold cross-validation. The result is shown in Figure S10.

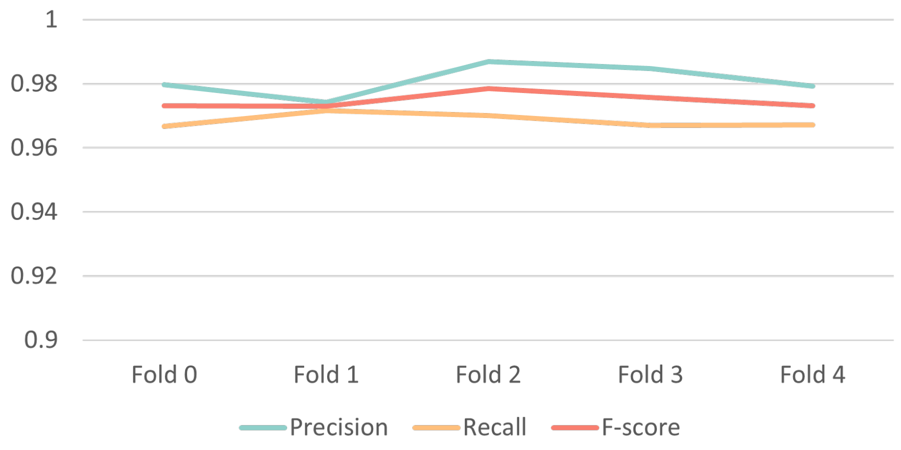

Figure S10: The performance of 5-fold cross-validation on the PLSDB dataset.

The performance of PLASMe on 5-fold cross-validation is very stable, indicating that the model's performance is not affected by different sets of training/ test data. And the overall performance (precision: 0.981±0.005, recall: 0.969±0.002, F-score: 0.974±0.002) is slightly better than that on the PLSDB test set (precision: 0.970, recall: 0.965, F-score: 0.967), which suggests that the date-based data partition method does not lead to an easier test set for the model.

11 Comparison between Transformer and LSTM

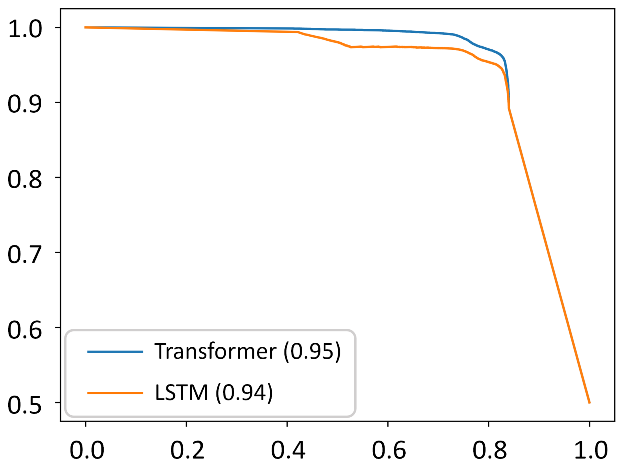

Figure S11: The ROC of Transformer and LSTM on the contig data sets. The area under the curve for Transformer and LSTM is 0.95 and 0.94, respectively.

Based on the design and applications of Transformer in other fields, the attention mechanism in Transformer gives it an essential advantage over LSTM in that Transformer can better capture long-range contextual relations. Through our experiments, we found that Transformer also outperforms LSTM on short sequences. In order to compare the performance of the Transformer and LSTM on short sequences, we trained an LSTM model using the PLSDB training set and compared its performance with the Transformer on the short contig test sets. The LSTM model consists of one bidirectional LSTM layer followed by two fully connected layers, with sizes of 64 and 1, respectively. The size of the word embedding is 512, and we set the hidden dimension in the LSTM unit to 2048.

We trained the model using a learning rate of 0.001, a batch size of 512, binary cross-entropy loss, and the Adam optimizer. To construct the dataset, we merge the contig 1K, contig 2K, and contig 3K test sets. We only used BLAST to assign the taxonomy of contigs, and then used LSTM and Transformer to predict the labels of query contigs. We plot the precision-recall curve and calculate the area under the curve. The comparison result is shown in Figure S11. We can see that Transformer outperforms LSTM slightly. No obvious advantage of using LSTM on short contigs is observed.

12 Comparing PLASMe and Recycler on the CAMI datasets

In addition to comparing PLASMe with learning-based and alignment-based tools, we also compare it with Recycler, a representative graph-based tool, on the CAMI2 datasets. Unlike PLASMe and other benchmarked tools that identify plasmids from provided contigs, graph-based tools assemble plasmids from a provided assembly graph. We used metaSPAdes to assemble the reads to obtain the assembly graph. Recall and precision were used to evaluate the results, as shown in equations 7 and 8.

$$\text{Recall} = \frac{\#\text{ of identified reference plasmids}}{\#\text{ of total reference plasmids}} \tag{7}$$

$$\text{Precision} = \frac{\#\text{ of correctly identified plasmid contigs}}{\#\text{ of identified putative plasmids}} \tag{8}$$

The recall is defined as the ratio of the identified reference plasmids to the total number of plasmid genomes in a sample. To obtain the number of identified reference plasmids, we aligned the identified putative plasmids to the reference plasmids. Then, we calculate the ratio of plasmids in the reference that can be aligned with an identity and coverage greater than 80%. And precision is defined as the ratio of correctly identified plasmid contigs to the returned plasmid contigs. The results are shown in Figure S12.

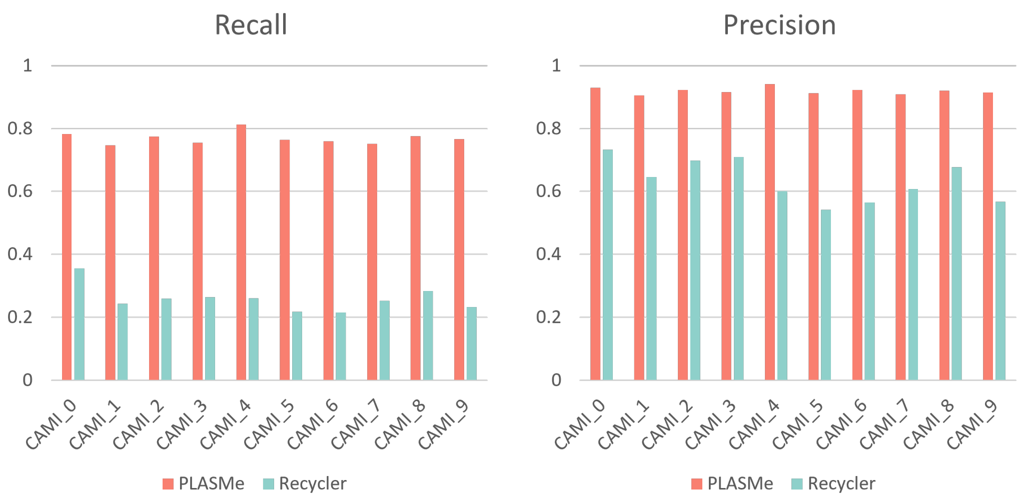

Figure S12: The recall and precision of PLASMe and Recycler on the 10 CAMI datasets.

PLASMe has higher recall and precision than Recycler on the CAMI datasets. As a graph-based approach, Recycler identifies putative plasmid sequences using the circularity and coverage from the assembly graph, which tends to be affected by the sequencing coverage of the plasmids and chromosomes. When the plasmids can only be partially assembled, the reliance on topological circularity can lead to a low recall. On the other hand, although Recycler can find novel plasmids, inconsistent chromosome coverage in complex samples leads to false positives, which is discussed in [3]. Alla et al. mentioned that graph-based tools are not well suited for metagenomic data because of the typically non-uniform chromosome coverage. In the original Recycler paper, the performance worsens when the sample becomes more complex or the coverage decreases. The CAMI

dataset contains many species, and most of them have low coverage, which may explain why the performance of the Recycler is not good on the CAMI datasets.

13 The intersection between different tools on the real data

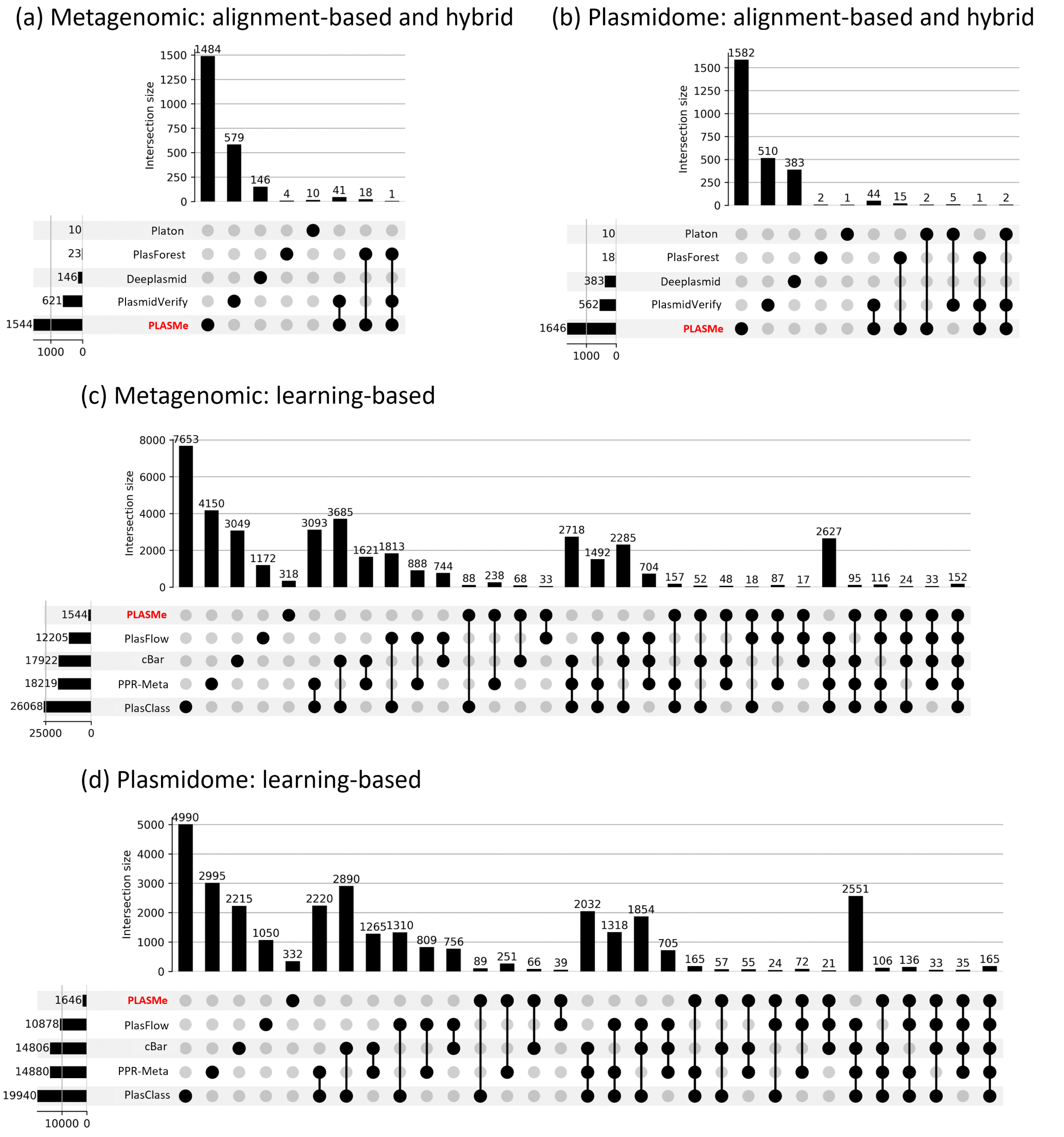

Figure S13: Intersection of the identified plasmids between different tools on the real metagenomic and plasmidome datasets

To compare the results of the different tools, we computed the intersection of the results between different tools, as shown in Fig. S13. To show the results more clearly, we divided the tools into two groups for analysis: a group of learning-based methods with higher recall and a group of reference-based and hybrid methods with higher precision. From Fig. S13 (a), S13 (b), we can see that PLASMe shared the highest number of contigs with other tools using alignment-based and hybrid methods (60 and 64 in metagenomic and plasmidome data, respectively) Based on the experimental results on CAMI datesets, the other four learning-based tools have low precision and introduce more

false positives. Although they have relatively large overlaps, as shown in Figure S13 (d), the overlap may contain many contigs from chromosomes.

14 Running time analysis of different tools

To show the reasons behind the significant differences in runtime among different tools, we will briefly outline the steps involved in running each tool. Tools cBar, PlasFlow, and PlasClass use k-mer frequency to construct feature vectors for machine learning prediction. Building the k-mer frequency vector is highly efficient. Mob-Recon, PlasForest, and PlasmidFinder rely mainly on alignment to identify plasmids or to construct feature vectors. Their runtime is mainly spent on running BLASTn in megablast mode, which is fast too. PlasmidVerify and Platon both use HMMER to check whether contigs contain the protein families in the database, which runs slower and therefore leads to longer runtime for these tools. Deeplasmid needs to derive multiple features on the sequences to construct the feature vector, including HMMER-generated protein domain alignment features. Additionally, it feeds the input to multiple models and uses the averaged prediction probabilities to minimize the error. Both factors contribute to the long runtime of Deeplasmid.

15 Method of extracting important tokens

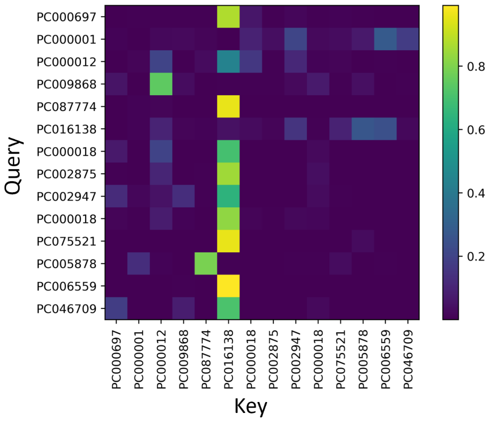

Figure S14: NC_011410.1's eighth head attention distribution.

To evaluate the token importance, we extracted the attention distribution of each head by feeding the sequence into the trained model. The attention distribution is calculated and normalized using $softmax(QK^T/d_k)$, where Q is query, K is key, and d_k is the dimension of K. As shown in Fig. S14, the rows in the attention distribution represent the query, and the columns represent the key. The larger the attention between a pair of query and key, the stronger the association between them. To evaluate the importance of tokens in the contig, we summed up the attention of all queries for each key. The key with the largest sum was treated as the most important token in the sequence. There were eight heads in the Transformer, and each head might learn a different attention distribution. So, we calculated the most important token for each head and measured importance by dividing the frequency of the token rated as the important token by the number of times it appears.

Here we choose the order containing the most plasmids, *Enterobacterales*, to interpret the Transformer. We inputted 16,432 *Enterobacterales* plasmids into the *Enterobacterales* model to evaluate the token importance. We only evaluated the tokens whose occurrence was at least 50.

16 The distribution of the average attention score of each token

In order to evaluate the significance of different tokens to the model, we also plotted the distribution of average attention scores. Some tokens exhibit very low occurrence, meaning that these tokens don't have much impact on the results. Thus, we selected tokens with frequencies exceeding ten times and computed their average attention scores. In the end, we analyzed the attention score distribution of 17,495 tokens, as shown in Figure S15.

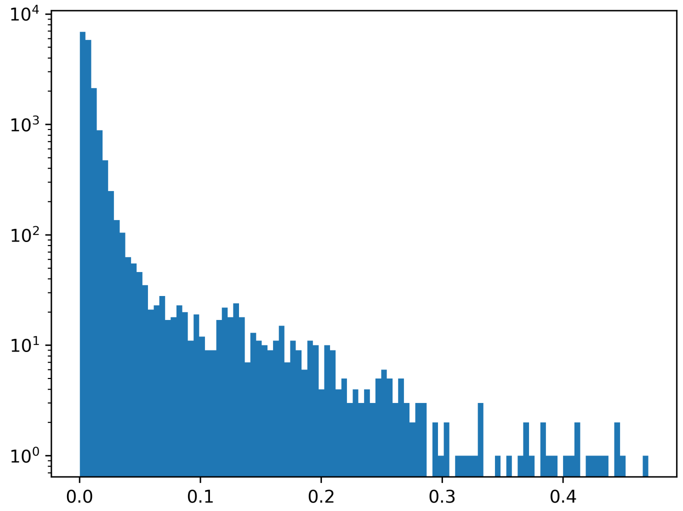

Figure S15: The histogram of the average attention scores of 17,495 tokens in *Enterobacterales*. X-axis: the average attention score. Y-axis: the number of tokens (scaled by the logarithm with a base of ten). In this plot, we used a bin width of 0.005, which resulted in some attention scores not including any tokens.

The distribution aligns with our expectations, as certain PCs are known to have core functions in plasmids, resulting in high attention scores and a decisive effect on plasmid identification. However, the majority of tokens do not have high average attention scores and may occur frequently but only contribute to specific sequences, resulting in a lower average attention score.

17 Predicting protein functions of the unknown tokens

Although we used multiple databases and tools to annotate the function of the top 50 important tokens, there are still 27 tokens that cannot be annotated with any function. To predict the functions of these unknown tokens, we used DeepFRI, which integrates the features from protein sequence and structure, to predict the functions. During the prediction, we used AlphaFold to predict the structure of the protein and then input the predicted structure into DeepFRI for prediction. To ensure the reliability of the results, we only predict the function for PCs with a length greater than 150 amino acids, as shown in Table S2.

Table S2: Summary of unknown tokens and the predicted functions.

PC token ID	Length (aa)	Function
41646	204	Iron binding
32278	177	Transmembrane
15827	233	Transmembrane

We can see that PC_{41646} is associated with iron binding. This protein may act as a virulence factor and enhance the pathogenicity of bacteria. For example, in bacteria whose hosts are eukaryotes, iron-binding proteins can help bacteria improve their ability to obtain iron in the environment, which is a critical factor for bacterial growth [4]. Meanwhile, PC_{32278} and PC_{15827} are both predicted to be transmembrane-related proteins. These two PC are likely related to the horizontal gene transfer of plasmids between bacteria.

18 Annotating the high-similarity regions on the plasmids

To annotate regions on plasmids sharing high similarity with chromosomes, we aligned complete bacterial and archaeal genomes in RefSeq to plasmids in PLSDB. Based on the alignment, we defined the high-similarity region as the alignment where the length was greater than 300 bp, and the identity was greater than 90%.

During testing, we will parse the alignment results and provide the alignment length and the start and end positions of the query contigs aligned to the high-similarity regions in the output file. Users can use the alignment results to determine whether the identified plasmids are likely to be false positives.

19 Supplementary Tables

Table S3: The hyperparameters used by AA, aa-BPE, and nt-BPE models. Except for the hyperparameters, the models with different tokens use the same model structure.

Tokens	Vocabulary size	Input len	Embedding size	Batch size	Loss	Optimizer	Learning rate	Dropout rate
AA	24	1000	128	512	BCE	Adam	0.001	0.1
aa-BPE	5002	350	512	512	BCE	Adam	0.001	0.1
nt-BPE	5002	350	512	256	BCE	Adam	0.001	0.1

Table S4: Details for data partition. *Date Cutoff* is the date cutoff for dividing the data into training and test sets. *Train* is the number of plasmids in the PLSDB training set. *Test* is the number of plasmids in the PLSDB test set. *Train/Test* is the ratio of training to test samples.

Order	Date Cutoff	Train	Test	Train/Test
Thermales	5/19/2021	46	12	3.83
Entomoplasmatales	3/2/2020	45	8	5.62
Deinococcales	3/23/2020	45	8	5.62
Neisseriales	9/25/2020	108	27	4.00
Bacteroidales	5/11/2021	102	23	4.43
Legionellales	7/5/2019	81	21	3.86
Synechococcales	12/30/2020	77	20	3.85
Cytophagales	6/7/2020	76	14	5.43
Mycoplasmatales	1/27/2019	74	14	5.29
Alteromonadales	3/7/2020	70	18	3.89
Spirochaetales	10/4/2018	411	100	4.11
Corynebacteriales	10/22/2020	355	87	4.08
Burkholderiales	8/4/2020	312	77	4.05
Xanthomonadales	2/9/2021	308	53	5.81
Campylobacterales	7/21/2020	280	68	4.12
Thiotrichales	11/28/2019	260	61	4.26
Vibrionales	6/26/2020	258	62	4.16
Aeromonadales	1/27/2021	240	55	4.36
Sphingomonadales	6/18/2020	229	57	4.02
Streptomycetales	12/6/2020	149	37	4.03
Nostocales	7/9/2020	116	36	3.22
Pasteurellales	6/4/2020	113	28	4.04
Lactobacillales	1/24/2021	2812	907	3.10
Bacillales	12/21/2020	2517	625	4.03
Pseudomonadales	9/27/2020	1572	356	4.42
Rhodobacterales	10/29/2019	566	140	4.04
Hyphomicrobiales	12/21/2020	529	130	4.07
Eubacteriales	12/15/2020	232	47	4.94
Rhodospirillales	4/3/2020	203	51	3.98
Micrococcales	8/18/2020	169	41	4.12
Enterobacterales	12/13/2020	13167	3265	4.03
Chlamydiales	6/2/2020	58	18	3.22
Chroococcales	9/29/2017	51	12	4.25
Flavobacteriales	8/4/2020	50	12	4.17
other	8/16/2019	770	184	4.18

Table S5: The commands used by different tools. Here, we use 'INFILE' for the input file, 'OUTPATH' for the path of the outputs, and 'NUM_THREAD' for the number of threads.

Tools	Commands
Deeplasmids	sudo docker run -it -v INFILE:/srv/jgi-ml/classifier/dl/in.fasta -v OUTPATH:/srv/jgi-ml/classifier/dl/outdir billandreo/deeplasmid.tf.gpu2 deeplasmid.sh in.fasta outdir
plasmidVerify	./plasmidverify.py -f INFILE -o OUTPATH –hmm Pfam-A.hmm -t NUM_THREAD
PlasClass	python classify_fasta.py -f INFILE -o OUTPATH -p NUM_THREAD
Platon	platon –output OUTPATH –threads NUM_THREAD INFILE
PlasFlow	PlasFlow.py –input INFILE –output OUTPATH
cBar	cBar.pl INFILE OUTPATH
PPR-Meta	PPR_Meta INFILE OUTPATH
MOB-recon	mob_recon -i INFILE -o OUTPATH -n NUM_THREAD
PlasmidFinder	plasmidfinder.py -p plasmidfinder_db -i INFILE -o OUTPATH
BLASTN	blastn -db blastn_db -outfmt 6 -out OUTPATH -num_threads NUM_THREAD
PlasForest	python PlasForest.py -i INFILE -o OUTPATH –threads NUM_THREAD

Table S6: Summary table of the tokens with known functions among the 50 most important tokens

Token ID	Database	Entry ID	Function Description	Score
28583	Pfam	PF13408	Recombinase zinc beta ribbon domain	0.474138
5879	Pfam	PF04365	Ribonuclease toxin, BrnT, of type II toxin-antitoxin system	0.472368
31617	Pfam	PF18607	ParA helix turn helix domain	0.432692
61778	Pfam	PF06892	Phage regulatory protein CII (CP76)	0.4125
45996	Pfam	PF10624	Plasmid conjugative transfer entry exclusion protein TraS	0.411765
10135	Gene3D	G3DSA:1.20.1250.20	MFS general substrate transporter like domains	0.379237
55539	Pfam	PF13610	DDE domain	0.375
34944	Pfam	PF03230	Antirestriction protein	0.370833
45248	Pfam	PF01266	FAD dependent oxidoreductase	0.363095
2780	Pfam	PF02371	Transposase IS116/IS110/IS902 family	0.358929
8519	Pfam	PF01548	Transposase	0.352848
52197	Pfam	PF03797	Autotransporter beta-domain	0.352273
41470	Pfam	PF09019	EcoRII C terminal	0.35
49791	Pfam	PF13561	Enoyl-(Acyl carrier protein) reductase	0.329545
3116	Pfam	PF07424	TrbM	0.320313
32059	Pfam	PF13117	Cag pathogenicity island protein Cag12	0.316176
7638	TIGRFAM	TIGR03474	incFII_RepA: incFII family plasmid replication initiator RepA	0.315549
3776	Pfam	PF00005	ABC transporter	0.315367
41393	Pfam	PF04134	DCC1-like thiol-disulfide oxidoreductase	0.31
48131	CDD	cd01139	TroA_f	0.308824
4312	Pfam	PF13610	DDE domain	0.304245
60329	CDD	cd03020	DsbA_DsbC_DsbG	0.295455
2624	TIGRFAM	TIGR00180	parB_part: ParB/RepB/Spo0J family partition protein	0.284722

References

[1] William J Youden. Index for rating diagnostic tests. *Cancer*, 3(1):32–35, 1950.

[2] Daniel N Baker and Ben Langmead. Dashing: fast and accurate genomic distances with hyperloglog. *Genome Biology*, 20:1–12, 2019.

[3] Alla L Lapidus and Anton I Korobeynikov. Metagenomic data assembly–the way of decoding unknown microorganisms. *Frontiers in Microbiology*, 12:613791, 2021.

[4] Manuela Di Lorenzo and Michiel Stork. Plasmid-encoded iron uptake systems. *Plasmids: Biology and Impact in Biotechnology and Discovery*, pages 577–597, 2015.
